# Comprehensive Proteomic Characterization of the Human Colorectal Carcinoma Reveals Signature Proteins and Perturbed Pathways

**DOI:** 10.1038/srep42436

**Published:** 2017-02-09

**Authors:** Jian-Jiang Hao, Xiaofei Zhi, Yeming Wang, Zheng Zhang, Zeyu Hao, Rong Ye, Zhijie Tang, Fei Qian, Quhui Wang, Jianwei Zhu

**Affiliations:** 1 Department of Gastrointestinal Surgery, Affiliated Hospital of Nantong University, Nantong 226001, China; 2 Poochon Scientific, Frederick, Maryland 21701, USA; 3 Whiting School of Engineering of Johns Hopkins University, Baltimore, Maryland 21218, USA

## Abstract

The global change in protein abundance in colorectal cancer (CRC) and its contribution to tumorigenesis have not been comprehensively analyzed. In this study, we conducted a comprehensive proteomic analysis of paired tumors and adjacent tissues (AT) using high-resolution Fourier-transform mass spectrometry and a novel algorithm of quantitative pathway analysis. 12380 proteins were identified and 740 proteins that presented a 4-fold change were considered a CRC proteomic signature. A significant pattern of changes in protein abundance was uncovered which consisted of an imbalance in protein abundance of inhibitory and activating regulators in key signal pathways, a significant elevation of proteins in chromatin modification, gene expression and DNA replication and damage repair, and a decreased expression of proteins responsible for core extracellular matrix architectures. Specifically, based on the relative abundance, we identified a panel of 11 proteins to distinguish CRC from AT. The protein that showed the greatest degree of overexpression in CRC compared to AT was Dipeptidase 1 (DPEP1). Knockdown of DPEP1 in SW480 and HCT116 cells significantly increased cell apoptosis and attenuated cell proliferation and invasion. Together, our results show one of largest dataset in CRC proteomic research and provide a molecular link from genomic abnormalities to the tumor phenotype.

Extensive genomic characterizations of human cancers have revealed cancer genome landscapes including a list of 140 candidate oncogenes and tumor suppressor genes, which are frequently mutated in tumors^1^,^2^,^3^. Alterations in these genes such as APC, p53 and KRAS, as well as in genes involved in the Wnt and TGF-β signaling pathways are considered as the most common initiating events of colorectal cancer (CRC)^4^,^5^. However, it is not fully understood how a few or a dozen of mutated tumor suppressor genes and oncogenes drive cancers^2^. A recent study of the proteogenomic characterization of CRC demonstrated that the mRNA transcript abundance did not reliably predict protein abundance differences between tumors^6^. This study reinforced the importance of measuring the protein abundance alteration in CRC. Although many studies have focused on measuring the protein changes associated with CRC^7^,^8^,^9^, a comprehensive characterization of the CRC proteome has not been accomplished. We thus analyzed proteomes of 44 samples (22 paired tumors and adjacent normal tissues) using a standardized quantitative proteomics workflow including pre-fractionation of protein samples by SDS-gel for maximizing the coverage, evaluation of the completeness of proteomic profiles using ten groups of well-defined “housekeeping” protein complexes for ensuring data quality, spectral counting-based quantification using the unit of parts per million (ppm) for describing the protein abundance, and a novel pathway analysis strategy for analyzing the biological consequence of the changes of the proteome. This strategy enabled us to generate a comprehensive map of the CRC proteome and to identify its abnormal features.

Membrane-bound Dipeptidase 1 (DPEP1, also known as microsomal dipeptidase or renal dipeptidase) is a zinc-dependent metalloproteinase that has been shown to process a plethora of peptides and antibiotics, as well as to be involved in the glutathione and leukotriene metabolism. The encoded protein is anchored to the membrane by a covalently attached glycosyl-phosphatidylinositol moiety and has a highly hydrophobic sequence located at its carboxyl terminus. In the current study, we investigated DPEP1 as a candidate marker in CRC. Our study not only provides a useful database for CRC biomarker discovery but also provides new insights into DPEP1-mediated cancer progression.

## Results

### Quantitative proteomic analysis reveals a significant pattern of changes in protein abundance in the CRC proteome

To quantify the changes in the CRC proteome, paired tumor and AT samples were processed and fractionated at the protein level by SDS–polyacrylamide gel electrophoresis and at the peptide level by basic reversed-phase liquid chromatography and analyzed on a high-resolution Fourier-transform mass spectrometer (Q-Exactive Orbitrap). Approximately 44 proteomic profiles were generated by analyzing 22 paired CRC and AT samples (Supplementary Tables 1, Supplementary Dataset 1 and 2). The integrity of the 44 profiles was assessed based on the coverage of ten groups of 444 well-known “housekeeping” proteins or complexes (Supplementary Dataset 3 and 4) and scored at an average of 92 of 100, suggesting that these profiles were comparable.

**Figure 1:**
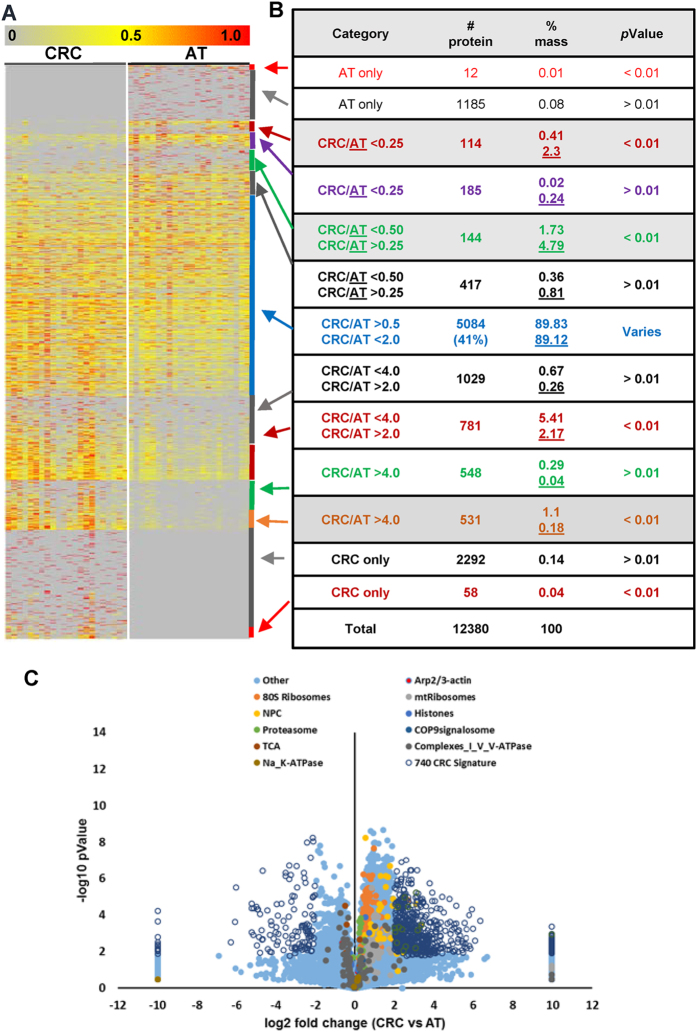
Proteomic characterization of the proteomes of CRC and AT. (**A**) A heat map depicting the relative abundance of 12,380 proteins identified across 44 samples (22 CRC and 22 AT). The color key indicates the relative abundance of each protein (0 to 1.0) across 44 samples. (**B**) A summary of statistical analyses of differentially expressed proteins (right, each color representing a category; ratio of CRC/AT was calculated based on the average of 22 samples). (**C**) Volcano plot demonstrating the fold change of protein abundance between CRC and AT. The x-axis represents the log2 of fold changes (CRC versus AT), and the y-axis represents the statistically significant p-value (−log10 of *p*-value, n = 22).

A total of 12,380 proteins were identified across 22 CRC and 22 AT samples, accounting for approximately 60% of the annotated proteins in the human genome and representing the accumulated analysis results of the existence of a human core proteome of approximately 10,000–12,000 ubiquitously expressed proteins. Among these, 8,832 proteins were detected in both CRCs and ATs, 10,030 proteins were detected in ATs, and 11,183 proteins were detected in CRCs (Fig. 1A and Supplementary Dataset 1 and 2).

**Figure 2:**
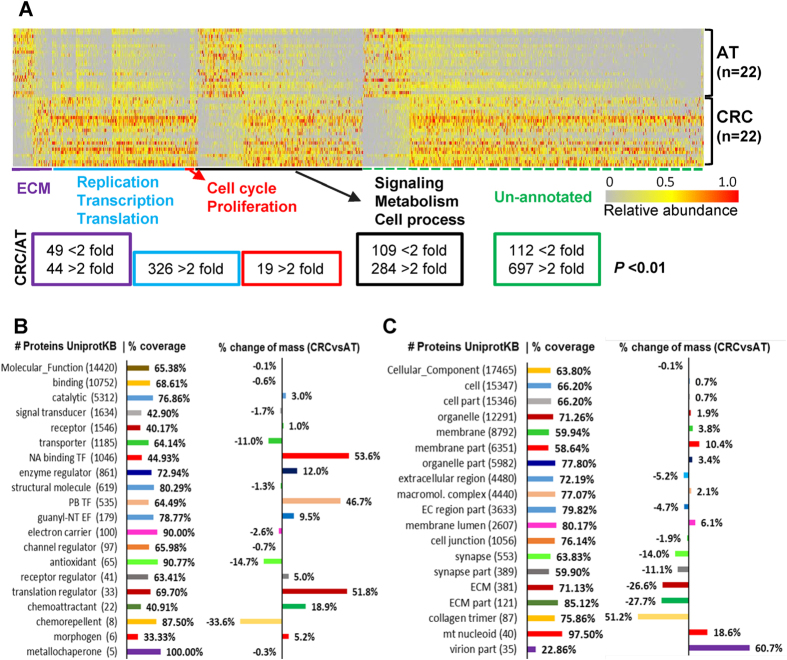
Summary of protein abundance variation in CRC and AT. (**A**) Heat map depicting the comparison of the relative abundance of 1,640 ranked individual proteins (fold change >2, *p* < 0.01, n = 22) between 22 paired CRCs and ATs according to classified annotations. (**B** and **C**) Summary of coverage and the grouped protein abundance changes according to the molecular function classification (14,420 protein entries) and cellular component classification (17,465 protein entries). The listed numbers of proteins for each classification were obtained from the UniProtKB website, and the full name for each class is listed in Supplementary Table 2.

The changes in abundance of 12,380 proteins are analyzed and summarized in Fig. 1 (Supplementary Dataset 1 and 2). According to the variations in abundance, the 12,380 proteins were divided into three groups. The first group represented 41% (5,084) of the identified proteins, which constituted approximately 89% of the total protein mass and exhibited changes of less than 2-fold. These proteins contained high-abundance housekeeping proteins, including histones, ribosomal proteins, metabolic enzymes and cytoskeletal proteins (Fig. 1C, Supplementary Dataset 2). The second group accounted for 5,656 proteins, which constituted approximately 1.5% of the total protein mass and were the least abundant. This group included 3,477 proteins, which were detected in either ATs or CRCs, and 2,179 proteins, which were detected in both ATs and CRCs. However, their abundance changes were not statistically significant (*p* > 0.01, n = 22) (Supplementary Dataset 2). The third group represented 12% (1640) of the identified proteins that were either overexpressed (83.5%) or decreased (16.5%) significantly by at least 2-fold in tumors (*p* < 0.01, n = 22), and these proteins contributed to approximately 8.7% and 9.4% of the total protein mass for CRCs and ATs, respectively (Fig. 2A, Supplementary Dataset 5). Interestingly, the 1,370 proteins that were overexpressed in CRCs are generally low in abundance and have reported regulatory functions for various cellular processes. In contrast, most of the 270 proteins that were decreased in CRCs are highly abundant and are involved in cellular architecture, metabolism and colorectal function. Approximately 715 of the 1,640 proteins were highly differentially expressed (fold change >4, *p* < 0.01) and were considered a CRC proteomic signature (Fig. 1C).

To further evaluate the relevance of the changes in the CRC proteome to defined protein classifications, we analyzed the summed protein abundance from each class of proteins based on UniProtKB classifications (14,420 entries classified as different molecular functions and 17,465 entries classified as different cellular components). The average coverage for all different classes was 67% and exhibited no apparent difference between CRC and AT (Fig. 2B,C, Supplementary Table 2). Interestingly, the summed protein abundances for several classes, including protein-binding transcription factors, nucleic acid binding transcription factors, and translation regulators, were significantly increased, whereas those for collagen trimers, extracellular matrix (ECM) components and extracellular matrices were decreased in CRC (Fig. 2B,C, Supplementary Table 2). These changes are consistent with the above individual protein analysis, which implies that cancer cells grow rapidly with a less stable structural architecture.

### The elevation of proteins required for cell proliferation in tumors

**Figure 3:**
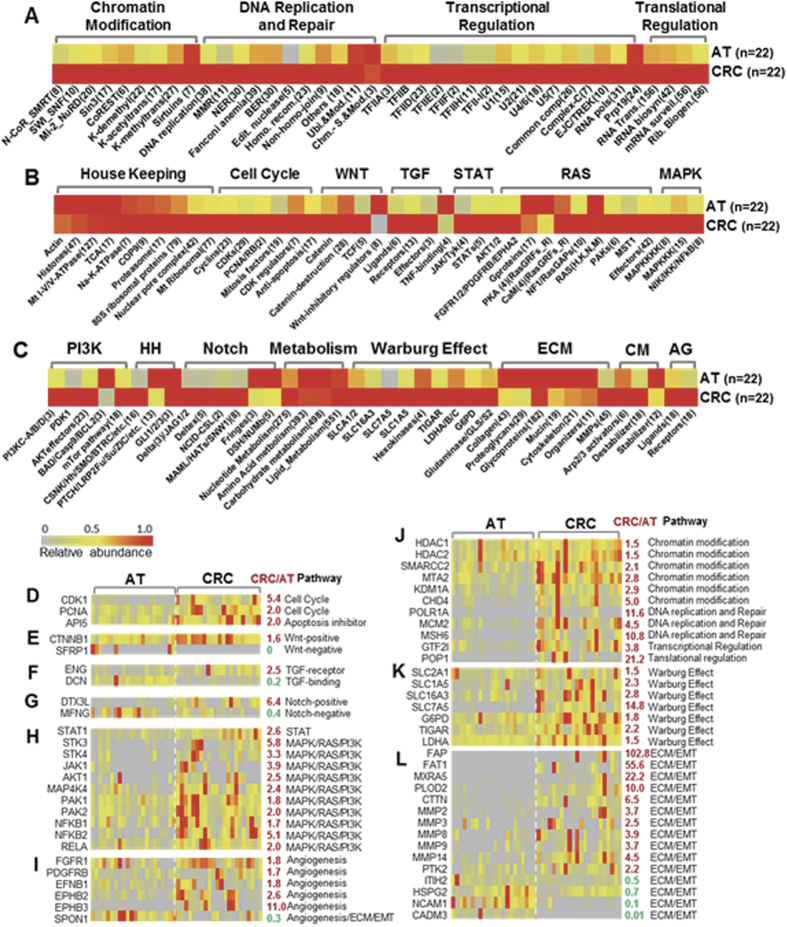
The hallmarks of the proteome transition illustrated by the differential expression of proteins or POCs in analyzed signaling pathways or cellular processes. (**A**–**C**) Heat map panels present comparisons of the relative abundance of pathway protein ontology chains (POC) at an average of 22 paired CRC and AT samples. The averages of 22 CRCs or 22 ATs are displayed as rows, and the POCs are displayed as columns. (**D**–**L**) Heat map panels depict comparisons of the relative abundance of selected individual proteins (rows) in analyzed pathways between 22 CRC samples and 22 AT samples (columns). The ratio of the average abundance of 22 CRCs to that of 22 ATs is highlighted in red (up) or green (down). The color key indicates the relative abundance of grouped proteins (0 to 1.0) at average (n = 22) between CRC and AT (**A**–**C**) or the relative abundance of individual proteins across 44 CRC and AT samples (**D**–**L**). ECM, extracellular matrix; HH, hedgehog; CM, cell motility; AG, angiogenesis. Note: Supplementary Fig. 3 is the full version of the heat maps of all pathways analyzed.

Cell cycle progression and DNA replication are central processes required for normal proliferation, development and homeostasis^10^. To test whether the proteins involved in these processes are subject to change in CRCs, the summed protein abundance for each pathway was analyzed. The summed abundance of proteins responsible for the cell cycle, including cyclins, CDKs, mitosis factors, proliferation regulators and anti-apoptosis regulators, increased by at least 50% in tumors, which is consistent with the view that increased anti-apoptosis and cell proliferation activities are often associated with tumor cells (Fig. 3B).

DNA replication machinery components were increased by greater than 100% in CRCs. The DNA damage repair complexes^11^, which include mismatch excision repair (MMR), nucleotide excision repair (NER), Fanconi anemia, editing and processing nucleases, base excision repair (BER), homologous recombination, non-homologous end joining, and other related components, were increased by at least 40% (Fig. 3A). This significant enrichment of the DNA replication machinery could be required for the high proliferation rate of tumor cells, and the augmented DNA damage repair machinery might be triggered by intensive DNA replication and transcription events.

### Elevation of chromatin modification

**Figure 4:**
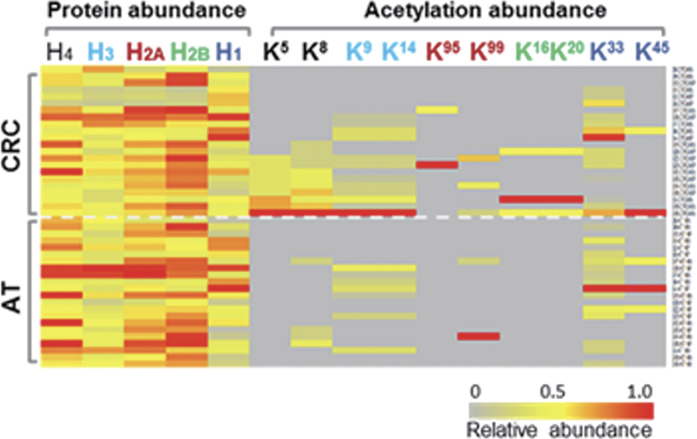
Comparison of the acetylation of lysines at the N-terminal tail of histones between CRC and AT. Approximately 22 CRC and 22 AT samples are displayed as rows, and the relative abundance of each histone protein or the relative abundance of acetylation sites are displayed as columns. The left panel presents histone proteins, with each histone labeled as a different color, and the right panel depicts acetylation sites for each histone protein, which are the same color as in the left panel. The color key indicates the relative abundance of proteins or the acetylation site (0 to 1.0) across 44 samples (22 paired CRCs and ATs).

The human chromatin modification complexes SWI/SNF, Mi-2/NuRD, CoREST, N-CoR/SMRT, and Sin3 as well as three types of enzymes, K-demethylases, K-acetyltransferases, and K-methyltransferases^12^, were significantly increased in CRCs (Fig. 3A); however, histone deacetylases exhibited minor changes. As shown in Fig. 3A, the transcription control machinery^13^, including the general transcriptional factors TFIIA, TFIIB, TFIID, TFIIE, TFIIF, TFIIH and TFII-I, RNA Pol I, II, and III and the RNA spliceosomes U1, U2, U4/6, U5, EJC/TREK, Complex-C, and spliceosome common components were also increased by greater than 50% in CRCs. Only the Prp19 complex components exhibited minor changes. These two predominant changes indicated that the intensive global chromatin modifications cooperated with heavy transcription events in CRC. Consistent with these observations, the proteins and enzymes involved in translation and nucleotide metabolism pathways were overexpressed in CRCs; however, the protein folding, sorting and degradation machinery was enriched moderately (Fig. 3A, Supplementary Fig. 3). To further confirm the enhanced activity in the genome of CRC, we studied the acetylation status of histone proteins, which commonly correlates with genome activity^14^,^15^. As shown in Fig. 4, the acetylation of lysine residuals on the tails of histones, especially H4, was increased in CRC, providing direct evidence for enhanced genome activity (Supplementary Fig. 4).

### Activation versus inhibition in signaling pathways

The inhibitory molecules of the WNT pathway, which include secreted frizzled-related proteins, Dickkopf-related proteins, and Kremen proteins, were decreased in CRCs (Fig. 3B). However, catenin and TCFs were increased in CRCs. TGF-β signaling molecules, including the ligands, receptors, and SMAD1-4, were enriched in CRCs. On the other hand, the TGF-β binding proteins CD109, decorin, dermatopontin, and TGF-β1-induced transcript-1 protein, which are known as negative regulators of cell proliferation^16^,^17^, were significantly decreased in CRCs.

A similar trend for the Notch and Hedgehog pathways was also observed (Fig. 3C, Supplementary Fig. 3). The inhibitory molecules Fringers and Numb in the Notch pathway were decreased in tumors. However, the Notch, Delta, gamma secretase complex (PESNEN, APH1A/B, PSEN1/2, Nicastrin and ADAMs), E3 ubiquitin-protein ligase, DTX3L, CSL, and co-activator SNW domain-containing protein-1 were increased in tumors. Similarly, Hedgehog signaling factors, including inhibitory molecules, such as PATCHs, Rab23, and the suppressor of fused homolog and cAMP-dependent protein kinases, were decreased in CRCs, whereas hedgehog, the smoothened homolog, and casein kinases were enriched.

### Variation in protein levels facilitates the Warburg glycolytic switch

We next analyzed the proteins involved in energy, carbohydrate, lipid, nucleotide, and amino acid metabolism, including 1,946 protein entries from the KEGG pathways database. Overall, proteins involved in energy and carbohydrate metabolism were slightly decreased, while those involved in lipid metabolism were slightly enriched in tumors (Fig. 3C, Supplementary Fig. 3). Proteins and enzymes involved in purine and pyrimidine metabolism were enriched by greater than 30% in CRCs versus ATs, whereas the overall levels of proteins and enzymes involved in amino acid metabolism were not increased in tumors. Significantly, important components^18^ involved in aerobic glycolysis, including glucose transporters and the key enzymes LDH-A, pyruvate kinase PKM, fructose-2,6-bisphosphatase TIGAR, and glucose-6-phosphate 1-dehydrogenase (G6PD), were remarkably increased, which suggested a shift of energy metabolism to Warburg aerobic glycolysis (Fig. 3C and K, Supplementary Fig. 3) and the reconstruction of a new metabolic state for sustained cell growth.

### Changes in core matrisome

The ECM provides structural and biochemical support to the surrounding cells and is a major component of the tumor microenvironment^19^. The “core matrisome” of the ECM, including collagens and proteoglycans, was significantly decreased by at least 40% in CRCs, except for glycoproteins, which exhibited minor changes (Fig. 3C and L). ECM-associated proteins, such as mucins, ECM organizers, and cytoskeleton-related proteins, were also decreased by at least 20%, whereas matrix metalloproteinases (MMPs) were increased by greater than 60% in tumors. The decrease of the core matrisome and the elevation of MMPs suggested that the normal ECM integrity and cell architecture were altered, which could create a favorable ECM environment for the abnormal growth and initiation of invasion and tumor cell metastasis. Consistent with this observation, many proteins promoting the EMT were overexpressed, whereas many proteins inhibiting EMT were decreased in tumors (Fig. 3L and Supplementary Fig. 3J). Angiogenesis^20^ is an important process that enables tumor growth. Consistent with this notion, receptors that activate angiogenesis were enriched in tumors, whereas SPON1, a vascular smooth muscle cell growth-promoting factor that inhibits angiogenesis, was significantly decreased in tumors (Fig. 3I, Supplementary Fig. 3H).

Cell motility is mainly controlled by actin cytoskeleton dynamics and is correlated with the ECM architecture. Rapid actin cytoskeletal remodeling in the cell cortex, a major contributor to tumor growth and metastasis, is regulated by upstream signaling regulators, including Arp2/3 activators, the Arp2/3 complex, actin polymerization regulators, actin filament (branched filament) stabilizers, and destabilizers^21^. Although the basic building materials, such as actin, myosin, and polymerization regulators, exhibited minor changes, the key regulatory components (PAKs and destabilizers) were significantly increased in CRCs (Fig. 3C, Supplementary Fig. 3B). In addition, PP1 regulatory subunits and stabilizers were decreased in CRCs, while Arp2/3 complex activators, Arp2/3 complex, and PP1catalytic subunits were mildly increased. These changes may reflect increased actin cytoskeleton dynamics and unstable cellular architectures, facilitating the invasion of tumor cells.

### Proteomic signature of CRC

**Figure 5:**
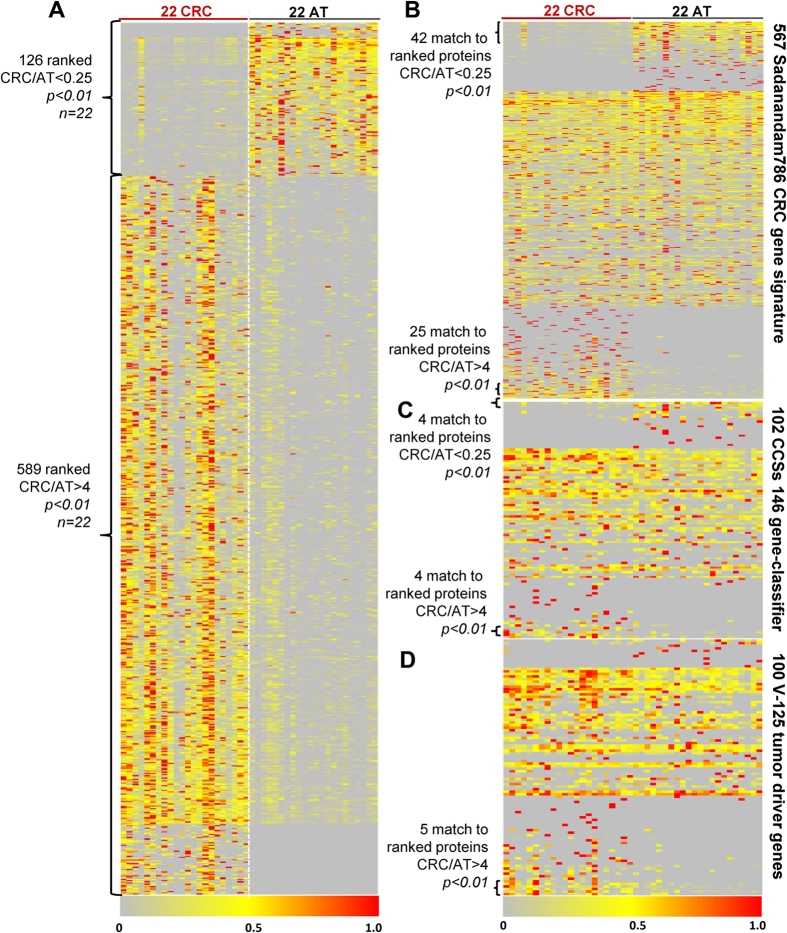
Identification of the CRC proteomic signature. Heat map depicting the relative abundance of ranked proteins (fold change >4, p < 0.01) identified across 44 samples (22 CRC and 22 AT). Twenty-two tumors and 22 AT samples are displayed as columns, and proteins are displayed as rows. The color key indicates the relative abundance of proteins (0 to 1.0) across 44 samples. The heat map presents the differential expression of (**A**) 715 CRC protein signatures across 44 samples, (**B**) 567 proteins (listed in Sadanandam 786 CRC assigner) identified across 44 samples, (**C**) 102 proteins (listed in Melo CCSs 146 gene classifier) identified across 44 samples, and (**D**) 100 proteins (listed in Vogelstein 125 tumor driver genes) identified across 44 samples. The details of the data is in Dataset 6.

Our quantitative proteomic analysis of 22 paired CRCs and ATs identified 740 significantly differentially expressed proteins (e.g. fold change >4, p < 0.01) (Fig. 5A). Among them 613 proteins had increased expression in all 22 cases of CRC patients (p < 0.01), while 127 proteins showed decreased expression (p < 0.01). Interestingly, although these 740 proteins encompassed about 6% of the total proteins identified, their mass was only 1.6% and 2.5% of the total mass in the CRCs and ATs, respectively. Most of the 127 proteins decreased in CRC but enriched in AT were high-abundant proteins, which were involved in cellular architectures, metabolisms and colorectal functions. In contrast, most of the 613 proteins enriched in CRC were low-abundant proteins, which were mostly involved in the regulation of cellular processes. This explained why the total mass of the 740 proteins was 58% more in AT than that in CRC.

To further evaluate the signature, we investigated if the ranked 740 proteins were overlapped with published CRC signatures. Sadanandam *et al*. identified a genomic data-based 786 CRC assigner for five CRC subtypes^22^. 567 proteins listed in the 786 CRC assigner were identified in this study (Fig. 5B). 136 of these proteins were differentially expressed by at least 1.5 fold in CRCs (p < 0.01) including 28 of them which were significantly overexpressed (CRC/AT > 4 fold, p < 0.01), and 42 which were significantly decreased (CRC/AT < 0.25 fold, p < 0.01). The 70 proteins were included in the 740 ranked protein list. 102 proteins listed in Melo’s 146 CRC gene classifier^23^ were also identified in this study. 23 of them were differentially expressed by at least 1.5 fold in CRC (p < 0.01), including 8 proteins that were changed by greater than 4 fold (p < 0.01) (Fig. 5C).

Many other studies had reported 1235 CRC signatures and they showed very limited overlap^23^. 962 proteins from this list were identified in this study. 225 of them were differentially expressed by at least 1.5 fold in CRC (p < 0.01), 55 proteins were overlapped with the 740 protein list. Additionally, we identified 28 well-known clinical or preclinical CRC biomarkers in this study (Fig. 5C and S10). Only Prominin-1 (CD133), a CRC stem cell marker, met with our specification as a CRC signature (>4 fold change, p < 0.01).

Vogelstein *et al*. summarized a list of 125 tumor driver genes^2^. 100 proteins from the list were identified in this study and 15 of them were differentially expressed by at least 1.5 fold in CRC (p < 0.01) (Fig. 5D), and 5 of them were overlapped with our ranked 740 proteins.

Overall, 1721 proteins, about 70% of the above CRC genes signatures and the tumor driver genes, were identified in this study, and about 20% of these identified proteins displayed differential expressions (>1.5 fold, p < 0.01). However, only 135 proteins were overlapped with our 740 CRC protein signature (fold change >4, p < 0.01, n = 22).

**Figure 6:**
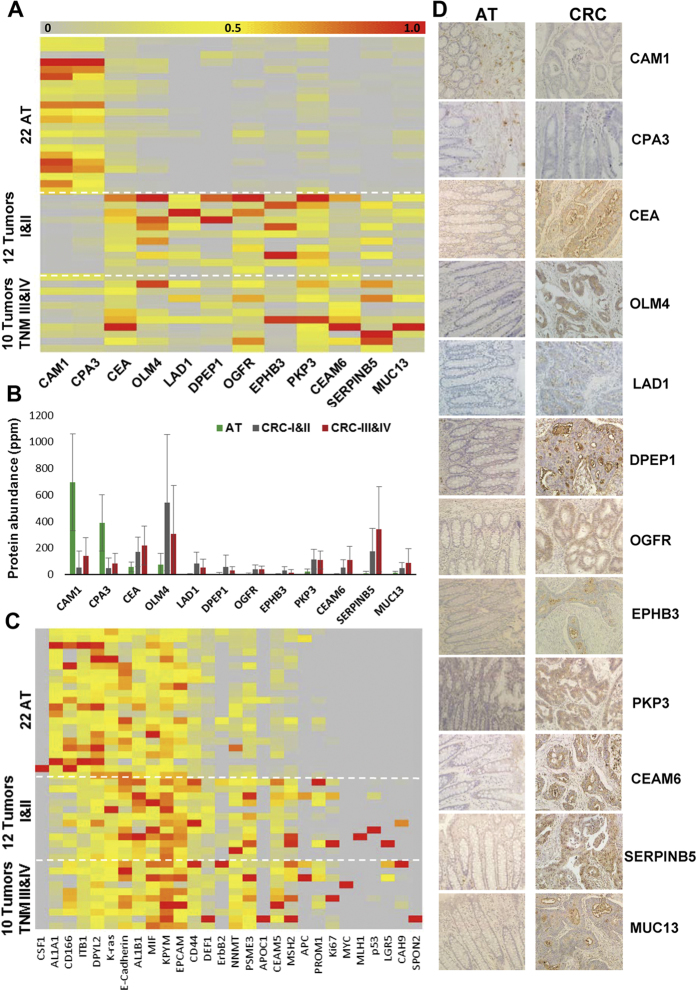
Biomarkers for human colorectal carcinoma. (**A**) Heat map showing differential expression of 11 protein biomarkers panel plus CEA across 22 AT, 12 TNM I&II tumors and 10 TNM III&IV tumors. The well-known CRC marker CEA was used as a comparison. It was not included in the 11 protein panel because its abundance change between CRCs and ATs was less than 4 fold. (**B**) Comparison of the average of protein abundance (ppm) of 12 biomarkers among 22 ATs, 12 TNM I&II tumors, and 10 TNM III&IV tumors. The average for AT based on 22 AT samples, for tumors based on 12 TNM I&II tumors and 10 TNM III&IV tumors respectively. (**C**) Heat map showing differential expression of well-known CRC biomarkers across 22 AT, 12 TNM I&II tumors and 10 TNM III&IV tumors. The colour key indicates the relative protein abundance (0 to 1.0) across 44 samples. Tumors and ATs are displayed as rows and proteins are displayed as columns. (**D**) Immunohistochemistry showing differential expression of the panel of 11 proteins and CEA in CRCs and ATs. Magnification of all images was ×200.

Considering the practical reality, a small panel of protein biomarkers would have more advantages. We identified a panel of 11 proteins based on the relative abundance (mean abundance in CRC >20 ppm) from the ranked 740 proteins to distinguish cancer tissues from normal colorectal tissues obviously (Fig. 6A–C). The well clarified tumor-associated gene CEA was used as a control gene. Two enzymes, mast cell carboxypeptidase A and chymase, secreted by mast cells, were significantly decreased in CRC. Nine proteins, were significantly overexpressed in cancer tissues. The abundance change of the 11 proteins was confirmed by immunohistochemistry assay (Fig. 6D).

### *In vitro* functional study of DPEP1

**Figure 7:**
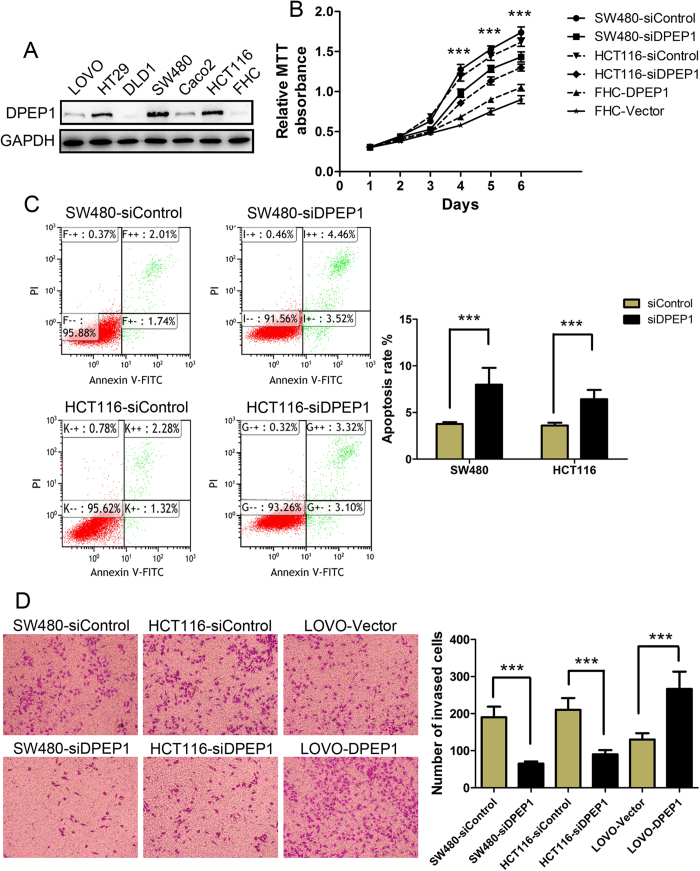
Functional study of DPEP1 in CRC cells. (**A**) Proteins prepared from CRC cell lines LOVO, HT29, DLD1, SW480, Caco2 and HCT116, and human colon normal epithelium cell line FHC were subjected to Western blot analysis. GAPDH was used as the loading control. After transfection with DPEP1 siRNA, Control siRNA, Vector plasmid and DPEP1-expression plasmid in the cells, (**B**) MTT viability assay, (**C**) Flow cytometry and (**D**) Transwell assay were used to detect the cell proliferation, cell apoptosis and cell invasion ability, respectively. ***P < 0.001.

Among the 11 identified proteins, Dipeptidase 1 (DPEP1) and Ladinin-1 (LAD1) were overexpressed in CRC with higher fold change (DPEP1, >1000 folds; LAD1, 188 folds). Given that LAD1 is a basement membrane protein and secreted by keratinocytes^24^, the up-regulation of LAD1 protein in CRC might be a secondary event. Thus we focused on the function of DPEP1 in CRC. To evaluate the functional roles of DPEP1 in CRC cells, we examined the effects of DPEP1 on cell proliferation, apoptosis and invasion (Fig. 7). CRC cell lines SW480, HCT116 and HT29 showed high DPEP1 expression levels, whereas human colon normal epithelium cell line FHC did not express DPEP1 protein (Fig. 7A). Transfection of SW480 and HCT116 cells with siRNA against DPEP1 resulted in a remarkable reduction of cell growth compared with control siRNA-transfected cells (Fig. 7B). Interestingly, overexpression of DPEP1 in FHC cells significantly enhanced cell proliferation (Fig. 7B). Flow cytometry revealed that siRNA silcecing of DPEP1 significantly increased cell apoptosis (Fig. 7C). In addition, siRNA-mediated silencing of DPEP1 significantly reduced the invasive ability of SW480 cells and HCT116 cells, and overexpression of DPEP1 in LOVO cells significantly enhanced the invasion ability (Fig. 7D). These results suggest that DPEP1 plays a vital role in CRC development.

## Discussion

Using a combination of comparative analysis of paired tumors and adjacent normal tissues and a novel algorithm of pathway analysis, we quantified the changes of the CRC proteome and identified the hallmarks of the proteome transition as important molecular events that occur during tumorigenesis. This discovery provides direct molecular evidence confirming the conceptual hallmarks of cancer, the acquired biological capabilities defined earlier^25^, and supports a model explaining how genomic alterations drive cancers. When molecular malfunction events, such as mutations in tumor driver genes, chromosomal instability and microsatellite instability, or environmental factors, occur and accumulate, a series of changes in gene expression may be initiated, resulting in the proteomic transition and eventually affording a selective growth advantage to the tumor cell. In addition, the approach we used demonstrates the feasibility of comprehensively measuring the changes of protein abundance in a human disease proteome and systematically analyzing their biological significances.

Proteomic studies of CRC have been conducted using surgically resected tissues to develop biomarkers and identify drug targets^26^,^27^. However, the reliable quantification of the changes in tumor proteome and the interpretation of a large proteomic data set remain challenging. The present CRC proteomic study was characterized by the use of Nanospray LC/MS/MS with a standardized quantitative proteomics workflow, including the maximal protein coverage through pre-fractionation of protein samples by SDS gel, the data quality control through evaluating the coverage of ten groups of well-defined “housekeeping” proteins, normalized spectral abundance factors (NSAFs)-based quantification to describe protein abundance, and a novel algorithm to perform quantitative pathway analysis.

In the present study, we achieved an analytical depth of 12380 proteins identified in 22 paired CRC and AT samples, to our knowledge the largest tumor proteome data set to date. In the subsequent analyses, we identified 715 significantly differentially expressed proteins (e.g. fold change >4, p < 0.01). Among them, 613 proteins had increased expression in all 22 cases of CRC patients, while 127 proteins showed decreased expression. To further confirm the signature, we also investigated if the ranked 715 proteins were overlapped with published CRC signatures. Sadanandam *et al*. identified a genomic data-based 786 CRC assigner for five CRC subtypes^22^. 567 proteins listed in the 786 CRC assigner were identified in our study and 70 proteins were included in our 715 ranked protein list. Moreover, 102 proteins listed in Melo’s 146 CRC gene classifier^23^ were also identified in this study. 962 proteins from De Sousa’s list^23^ were identified in our study and 55 proteins were overlapped with the 715 protein list. Vogelstein *et al*. summarized a list of 125 tumor driver genes^2^. 100 proteins from the list were identified in our study and 5 of them were overlapped with our ranked 715 proteins. The partial overlap of potential biomarkers between our study and previous reports supports the feasibility of our standardized quantitative proteomics workflow.

Of note, among the upregulated proteins in CRC tumor tissues were low abundant proteins which are responsible for malignant biological capabilities. For instance, DNA replication machinery components were increased by greater than 100% in CRC, and DNA damage repair complexes were increased by at least 40%. Cancer has long been associated with histone acetylation, which is known to enhance the transcription activity^28^. Our results showed the acetylation of histones especially H4 was increased in CRC, indicating that DNA replication cooperating with chromatin modifications contributed to the high genome activity in CRC. Deregulation of actin cytoskeleton and ECM-associated proteins were observed in CRC, which is consistent with previous studies^29^.

In contrast, among the down-regulated proteins in CRC tumor tissues were high abundant proteins which are responsible for cellular architecture, metabolism and colorectal function. The ECM can provide structural and biochemical support to cells and maintain cell polarity^30^. In the present study, the core components including collagens and proteoglycans were significantly decreased in CRC, as well as ECM-associated proteins, such as mucins, ECM organizers, and cytoskeleton-related proteins. The notion that altering normal ECM integrity and cell architecture could promote cancer cell invasion, which was consistent with our results, led to an increased interest in EMT process. As expected, many proteins inhibiting EMT were decreased in CRC, whereas EMT-promoting proteins were elevated.

Specifically, based on the relative abundance (mean abundance in CRC >20 ppm), we identified a panel of 11 proteins from the ranked 715 proteins to distinguish cancer tissues from normal colorectal tissues obviously. Among the 11 identified proteins, Dipeptidase 1 (DPEP1) and Ladinin-1 (LAD1) were overexpressed in CRC with higher fold change (DPEP1, >1000 folds; LAD1, 188 folds). Given that LAD1 is a basement membrane protein and secreted by keratinocytes^24^, the up-regulation of LAD1 protein in CRC might be a secondary event. Thus we focused on the function of DPEP1 in CRC. Membrane-bound DPEP1 is a zinc-dependent metalloproteinase that has been shown to process a plethora of peptides and antibiotics, as well as to be involved in the glutathione and leukotriene metabolism^31^,^32^. DPEP1 has been identified as a prognostic gene of colorectal cancer^33^. In the current study, we showed that DPEP1 was overexpressed in CRC, and knockdown of DPEP1 in SW480 and HCT116 cells significantly increased cell apoptosis and attenuated cell proliferation and invasion. Our study not only provides a useful database for CRC biomarker discovery but also provides new insights into DPEP1-mediated cancer progression.

In conclusion, we used a proteomics-driven approach to provide a comprehensive view of the CRC tissue proteome. This study provides insights into the deregulated proteins in CRC tumors that could act as likely drivers of CRC onset and progression, and may serve as potential CRC markers.

## Methods

### Tumor and adjacent tissue samples

All specimens were collected from patients from the Affiliated Hospital of Nantong University (Nantong, China) and Peking Union Medical College Hospital (Beijing, China) in accordance with approved human subject guidelines authorized by the Medical Ethics and Human Clinical Trial Committee at the hospitals. Written informed consents were obtained from all subjects. Following surgery, the tumor and adjacent normal tissue (AT) specimens were collected in separate tubes, maintained on dry ice during transportation, and stored at −80 °C before further processing. Twenty-two pairs of cancerous and adjacent normal tissue specimens were collected from 22 individual patients (Supplementary Table 1). AT specimens were obtained from the distal edge of the resection at least 5 cm from the tumor. A panel of known differentially expressed proteins was used to confirm the AT specimens (Supplementary Fig. 2). All CRC patients had histologically verified adenocarcinoma of the colon or rectum that was confirmed by pathologists. Patient characteristics were obtained from pathology records. Subjects with a history of other malignant diseases or infectious disease or who underwent surgery 6 months prior to the start of this research were excluded from this retrospective study.

### Preparation for protein extraction, separation of proteins, and in-gel trypsin digestion

Total protein extraction from fresh frozen tissue specimens was prepared using the following methods. Frozen tissue samples (0.05–0.1 gram) were cut into small pieces (1 mm size) using a clean sharp blade and transferred to 1.5-ml tubes. A 0.4-ml quantity of lysis buffer (20 mM Tris-HCl, pH 7.5, 150 mM NaCl, 1 mM Na_2_EDTA, 1 mM EGTA, 1% Triton X-100, and protease inhibitor cocktail pill) was added to each sample tube. The tissues were homogenized using a Dunce homogenizer. After homogenization, 50 μl of 10% SDS and 50 μl of 1 M DTT were added to the mixture followed by incubation at 95 °C for 10 min. After incubation, the extraction was sonicated to further break down the DNA. Sonicated mixtures were centrifuged at 15,000 × *g* for 10 min. Supernatants were collected and stored at −80 °C for further analysis. The protein concentration of the supernatants was determined with a BCA™ Reducing Reagent compatible assay kit (Pierce, Grand Island, NY, USA).

Equal quantities of protein (133 μg) from each sample were loaded onto a NuPAGE 4–12% Bis-Tris Gel (Life Technologies Corporation, Grand Island, NY, USA). After electrophoresis, the gel was stained with Simply Blue Safe Stain (Life Technologies Corporation) and subsequently de-stained. To prepare in-gel trypsin-digested peptides, the de-stained gel was washed with ion-free water thrice, and each lane representing one sample was sliced horizontally into 16 slices. Each slice was diced into tiny pieces (1–2 mm) and placed into 1.5-ml centrifuge tubes. Proteins in the gel were treated with DTT for reduction, followed by iodoacetamide for alkylation and further digestion with trypsin in 25 mM NH_4_HCO_3_ solution. The digested protein was extracted as described elsewhere. The extracted peptides were dried and reconstituted in 20 μl of 0.1% formic acid before nanospray LC/MS/MS analysis was performed.

### Nanospray LC/MS/MS analysis

Sixteen tryptic peptide fractions from one specimen sample were analyzed sequentially using a Thermo Scientific Q-Exactive Hybrid Quadrupole-Orbitrap Mass Spectrometer (Thermo Electron, Bremen, Germany) equipped with a Thermo Dionex UltiMate 3000 RSLCnano System (Thermo Dionex, Sunnyvale, CA, USA). Tryptic peptide samples were loaded onto a peptide trap cartridge at a flow rate of 5 μl/min. The trapped peptides were eluted onto a reversed-phase 25-cm C18 PicoFrit column (New Objective, Woburn, MA) using a linear gradient of acetonitrile (3–36%) in 0.1% formic acid. The elution duration was 110 min at a flow rate of 0.3 μl/min. Eluted peptides from the PicoFrit column were ionized and sprayed into the mass spectrometer using a Nanospray Flex Ion Source ES071 (Thermo) under the following settings: spray voltage 1.6 kV and capillary temperature 250 °C. The Q Exactive instrument was operated in the data-dependent mode to automatically switch between full scan MS and MS/MS acquisition. Survey full scan MS spectra (m/z 300–2,000) were acquired in the Orbitrap with 70,000 resolution (m/z 200) after the accumulation of ions to a 3 × 10^6^ target value based on predictive AGC from the previous full scan. Dynamic exclusion was set to 20 s. The 12 most intense multiply charged ions (z ≥ 2) were sequentially isolated and fragmented in the Axial Higher Energy Collision-induced Dissociation (HCD) cell using normalized HCD collision energy at 25% with an AGC target of 1e5 and a maximum injection time of 100 ms at 17,500 resolution.

### LC/MS/MS data analysis

The raw MS files were analyzed using the Thermo Proteome Discoverer 1.4.1 platform (Thermo Scientific, Bremen, Germany) for peptide identification and protein assembly. For each specimen sample, 16 raw MS files obtained from 16 sequential LC-MS analyses were grouped for a single database search against the Human UniProtKB human protein sequence databases (20,597 entries, 12/20/2013) based on the SEQUEST and percolator algorithms through the Proteome Discoverer 1.4.1 platform. The carbamido methylation of cysteines was set as a fixed modification. The minimum peptide length was specified as 5 amino acids. The precursor mass tolerance was set to 15 ppm, whereas the fragment mass tolerance was set to 0.05 Da. The maximum false peptide discovery rate was specified as 0.01. The resulting Proteome Discoverer Report contains all assembled proteins (a proteome profile) with peptide sequences and matched spectrum counts.

### Publically available MS raw data files used to evaluate the methods

Ninety-four sets of MS raw data files from The Cancer Genome Atlas (TCGA) CRC cancer program and 12 sets of MS raw datasets from the TCGA breast cancer program were downloaded from https://cptac-data-portal.georgetown.edu/cptacPublic/. Four sets of MS raw datasets from the CHPP proteome were downloaded from http://dx.doi.org/10.6019/PXD000529/.

### Protein quantification

The relative abundance for each identified protein in each proteome profile was calculated using the normalized spectral abundance factors (NSAFs) method^34^,^35^. To quantitatively describe the relative abundance, ppm (parts per million) was chosen as the unit with a total 1,000,000 ppm assigned to each proteome profile and was calculated based on its normalized NSAF.

The ppm (part per million) was calculated as follows:


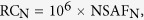


where

RC_N_ is the relative concentration of protein N in the proteome of the test sample;

NSAF_N_ is the protein’s normalized spectral abundance factor; and

N is the protein index.

Normalized spectral abundance factors (NSAFs) were calculated as follows:


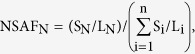


where

N is the protein index;

S_N_ is the number of peptide spectra matched to the protein;

L_N_ is the length of protein N (number of amino acid residues); and

n is the total number of proteins in the input database (proteome profile for one specimen sample).

The MEAN, STDEV, and paired T-test values (p-values) were calculated using Microsoft Excel. For the lowest abundant proteins the Fisher Exact Test p values were calculatedby analysis of detectable or undetectable of these proteins in 22 CRCsversus 22 ATs. The ratio of CRC versus AT was defined as 1,000 or 0.001 if the protein was not identified in AT or CRC samples, respectively.

To evaluate the ppm quantification method, we compared the ppm variation ranges among of subunits of four complexes with published Beck’s copy numbers data^36^. The relative protein abundance in ppm was calculated based on NSAFs and was compared with the published relative abundance calculated in Beck’s copy number. All subunits from four housekeeping protein complexes, including the Arp2/3 complex (7 subunits plus one isoform), the COP9 complex (8 subunits plus one isoform), and the proteasome (17 subunits) and TCA 17 enzymes, were used for comparison. As shown in Figure S6, the dynamic range of relative abundance among the members of a complex quantified using ppm was considerably reduced compared to that of Beck’s copy number. The dynamic range between the minimum and maximum for the four tested complexes exhibited 4- to 19-fold differences according to spectrum count-based measurements, whereas 9- to 600-fold differences were noted according to the published Beck’s copy number. This comparison indicated that the relative protein abundance measured based on spectrum count quantification would be closer to the real situation and that the housekeeping protein complexes could be used as the parameter to evaluate the “quality” of a proteome profile generated thereof.

### Evaluation of the comparative proteomic profiling workflow

The standardized spectral counting-based label-free quantitative proteomics workflow (Supplementary Fig. 1) described above was evaluated by examining the coefficients of variation (CVs). The CVs caused either by the LC-MS/MS system or by sample preparation were determined. The CVs caused by the inherent LC-MS/MS system (system error) included the nanoLC separation and the mass spectrometry measurement stability along with any potential inconsistencies related to the bioinformatic extractions of peptides by the proteome discoverer software. To define the CV caused by different LC/MS/MS runs, a set of 16 fractions prepared from a sample were run sequentially in duplicate. Two independent experiments were performed. As shown in Figure S7, the CV caused by the LC-MS system varied in reference to the relative concentration of proteins identified. The LC-MS/MS system caused the CV for the higher abundance proteins to be considerably reduced compared with the CV of the less abundant proteins. For example, the CV was less than 5% if the relative abundance of identified proteins was more than 1,000 ppm, but the CV was near 78% if the relative concentration of identified proteins was between 1 to 10 ppm due to the limitation of the mass spectrometer in detecting low-concentration peptides from a mixture. The average CV for all identified proteins between two independent analyses was 48% ± 27%. Because the lowest abundant proteins were mostly identified by either one independent analysis, the average CV for these proteins (<1 ppm) was increased (128% ± 40%). The average CV for all proteins with >1 ppm, which represented greater than 90% of identified proteins, was 28% ± 24%. Next, we evaluated the variation caused by sample processing by analyzing the same sample processed in triplicate. The average CV for proteins >1 ppm was 42.2% ± 27.5%. Given that system variation was inherent and independent of the sample, the CV caused by sample processing could be deduced by subtracting the system CV. Manual sample processing caused an average of 15% CV. In all analyses, the CV values and change patterns were similar, indicating that the proteomic workflow was reliable and repeatable.

### Evaluation of the “quality” of proteome profiles

Due to instrument limitations and the wide dynamic range of protein abundances, the most current LC/MS/MS settings were unable to recover the entire proteome, particularly the lowest abundance proteins in one experiment. Hence, development of an approach allowing evaluation of the integrity of a set of proteome profiles in an unbiased way is highly desirable. To achieve this goal, we focused on ten groups of well-characterized “housekeeping” protein complexes with the assumption that these proteins are essential for all live cells and that their detections would serve as an internal quality control for a set of proteomic profiles. Ten groups of well-known “housekeeping” protein complexes consisting of 444 proteins, including 359 unique proteins and 85 isoforms or subtypes (Supplementary Dataset 3), included the Arp2/3 complex (8 subunits plus alpha and beta actins), 86 (79 and 7 isoforms) cellular (60S and 40S) ribosomal proteins, 77 mitochondrial (28S and 39S) ribosomal proteins, nuclear pore complex 42 (38 subunits, GTP-binding nuclear protein Ran, Ran GTPase-activating protein 1 (RAGP1), Ran-specific GTPase-activating protein (RANG), Ran-binding protein 3 (RANB3)), 5 histones (H1 (9 subtypes), H2A (17 subtypes), H2B (17 subtypes), H3 (5 subtypes) and H4), proteasome complex (17 subunits), COP9 signalosome complex (9 subunits), TCA enzymes (17 key enzymes), mitochondrial respiratory chain complexes I–V (102 subunits), V-type proton (ATPase complex, 14 subunits consisting 24 isoforms), and Na+/K+-ATPase (sodium-potassium pump, 2 subunits, 7 isoforms). A score (0 to 100) was assigned based on the percentage of the 444 “housekeeping” proteins identified in a given profile. On average, 44 proteome profiles generated in this study were scored at 92, suggesting a consistent quality with all these profiles. To demonstrate the feasibility of this evaluation method, we assessed two sets of publically available MS raw data files (http://proteomics.cancer.gov/). One set of 94 MS raw data files (94 CRC samples) from the TCGA-CRC cancer program was scored at an average of 80.3. An additional set of 12 MS raw data files from the TCGA-breast cancer program was scored at an average of 98.5.

We next assessed the quality of a proteome profile based on the distribution of its protein population. The distribution of identified proteins per concentration range was analyzed using the Excel-histogram function. The average abundance for each identified protein was calculated as described above. The distribution of all identified 12,380 proteins was normal, with a major peak and a minor peak representing two populations. The major peak represented 62% (CRC) and 60% (AT) of identified proteins with a relative abundance greater than 1 ppm, and the minor peak represented approximately 38% (CRC) and 40% (AT) of identified proteins with an abundance less than 1 ppm. The majority of proteins in the minor peak were randomly identified with one or a few PSM across 22 CRC samples or 22 AT samples (Supplementary Dataset 1). To further evaluate the method, 94 sets of MS raw profiles from the TCGA-CRC cancer program, 12 sets of MS raw datasets from the TCGA-breast cancer program, and 4 sets of MS raw datasets from CHPP program were analyzed. The distributions of identified proteins in these studies exhibited the same normal distribution patterns (Supplementary Fig. 5, Supplementary Dataset 7).

### Pathway analysis

The cell’s functions are executed and regulated by the entire set of proteins (the proteome). The regulation of different cellular functions has been categorized into a number of pathways, such as the Wnt signaling pathway and the TGF signaling pathway. In each pathway, the components are generally named according to their function, including ligands, receptors, activating regulators, inhibitory regulators, and effectors. To measure the activation strength of a pathway, the protein molecules that belong to ligands, receptors, activating regulators, or inhibitory regulators were grouped as the pathway protein ontology chain (POC), and their relative abundances (ppm) were summed. Based on the summed abundance of each POC, the activation strength or activation status of a pathway could be compared between two proteome profiles. The proteins listed for all analyzed pathways and processes were obtained from the KEGG pathway database (http://www.genome.jp/kegg/pathway.html), and their functional annotation was manually confirmed using the UniProtKB protein database and the NCBI protein database or available publications. The proteins for all analyzed pathways are listed in Supplementary Dataset 3.

### Proliferation assay

Cells (2000/well) were seeded into 96-well plates and stained at the indicated time point with 100 μl sterile 3-(4,5-dimethythiazol-2-yl)-2,5-diphenyl tetrazolium bromide (MTT; Sigma, St. Louis, MO, USA) dye (0.5 mg/ml) for 4 h at 37 °C, followed by removal of the culture medium and the addition of 150 μl dimethyl sulfoxide (Sigma). The absorbance was measured at 570 nm, with 655 nm used as the reference wavelength. All tests were performed in triplicate.

### Apoptosis assay

Cells were stained with Apoptosis Detection Kit (Becton Dickinson, Franklin Lakes, USA) according to the manufacturer’s instructions. Cells were then tested on a FACScan flow cytometer (Becton Dickinson, Franklin Lakes, USA). All tests were performed in triplicate.

### Invasion assay

Invasion assays were performed with 24-well BioCoat Matrigel Invasion Chambers (BD) according to the manufacturer’s instructions. Briefly, 5 × 104 cells were seeded into 8 μm pore inserts in triplicate wells and incubated for 24 h. The invaded cells in lower filters were fixed in methanal and stained in crystal violet (Sigma) followed by counting under microscope. The data are to be expressed as the percent invasion through the Matrigel Matrix and membrane relative to the migration through the Control membrane (not coated with matrigel). All tests were performed in triplicate.

### Construction of small interfering RNA and recombinant plasmids

For the gene knockdown of DPEP1, small interfering RNA was synthesized by Invitrogen (Thermo Fisher Scientific Inc., Waltham, MA). SW480 cells and HCT116 cells were transfected with DPEP1-specific siRNA (sense: GGAGGUUCUUCUACUCGCCtt, antisense: GGCGAGUAGAAGAACCUCCtt) and control siRNA using Lipofectamine RNAiMAX reagents (Invitrogen, Grand Island, NY) according to the manufacturer’s protocol.

For the construction of DPEP1 recombinant plasmid, pcDNA3.1 (Invitrogen, Shanghai, China) was used. The full-length ORF of DPEP1 (1236 bp, NM_001128141.2) was amplified from cDNA of SW480 cells. The primers were as follows: forward, EcoRI-5′-AGAGAATTCATGTGGAGCGGATGGTGGCT-3′; reverse, BamHI-5′-AGAGGATCCAGTGTCCTCTCTGTCTGTCT-3′.

### Statistical analysis

Each experiment was repeated at least three times throughout the study. Data were reported as the mean ± SD. Statistical analysis was performed with SPSS software (SPSS Standard version 13.0; SPSS, Chicago, IL). P-value < 0.05 was considered statistically significant.

## Additional Information

**How to cite this article:** Hao, J.-J. *et al*. Comprehensive Proteomic Characterization of the Human Colorectal Carcinoma Reveals Signature Proteins and Perturbed Pathways. *Sci. Rep.* **7**, 42436; doi: 10.1038/srep42436 (2017).

**Publisher's note:** Springer Nature remains neutral with regard to jurisdictional claims in published maps and institutional affiliations.

## Supplementary Material

Supplementary Dataset 1

Supplementary Dataset 2

Supplementary Dataset 3

Supplementary Dataset 4

Supplementary Dataset 5

Supplementary Dataset 6

Supplementary Dataset 7

Supplementary Information
