# Supplementary material for: Comprehensive Proteomic Characterization of the Human Colorectal Carcinoma Reveals Signature Proteins and Perturbed Pathways
Source: Sci Rep. 2017 Feb 9;7:42436. doi: 10.1038/srep42436 (PMC5299448; doi:10.1038/srep42436)
Supplement: Supplementary Information [file srep42436-s8.doc]

**Comprehensive Proteomic Characterization of the Human Colorectal Carcinoma Reveals Signature Proteins and Perturbed Pathways**

Jian-Jiang Hao, Xiaofei Zhi, Yeming Wang, Zheng Zhang, Zeyu Hao, Rong Ye, Zhijie Tang, Fei Qian, Quhui Wang, and Jianwei Zhu

**Supplementary Figure 1**


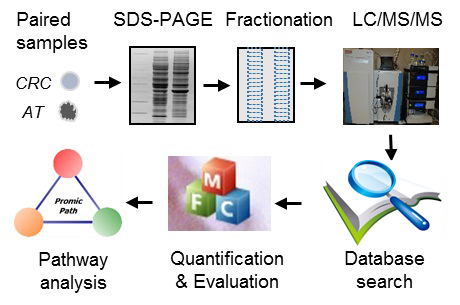


**Supplementary Fig. 1. A standardized mass spectrometry-based quantitative proteomic profiling and pathway analysis workflow.** The workflow included the preparation of cell lysate, protein separation by SDS-gel, and gel fractionation followed by in-gel digestion. LC MS/MS was performed on an Ultimate 3000 RSLCnano system coupled with a Q-Exactive mass spectrometer. Database searching and protein identification were performed using the Thermo Proteome Discoverer 1.4.1 platform. Label-free quantification was based on peptide spectrum match counts, and quantitative pathway analysis was performed as described in the Materials and Methods.

**Supplementary Figure2**

**
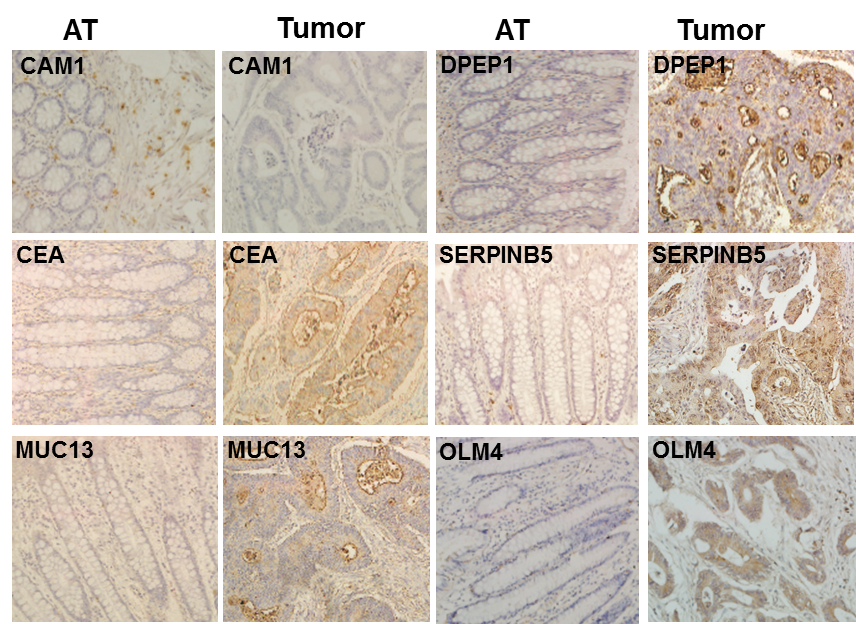
**

**Supplementary Fig.2. Confirmation of AT in comparison with CRC tumor tissue.** Immunohistochemistry depicting the differential expression of a panel of known CRC protein biomarkers.The enzyme mast cell carboxypeptidase A, which is secreted by mast cells, was used as the positive marker for normal colorectal tissue, and 5 known CRC protein biomarkers, DPEP1, CEA, SERPIN-B5, MUC13, and OLM4, were used in the determination of CRC. The magnification of all images was ×200.

**Supplementary Figure 3**

**
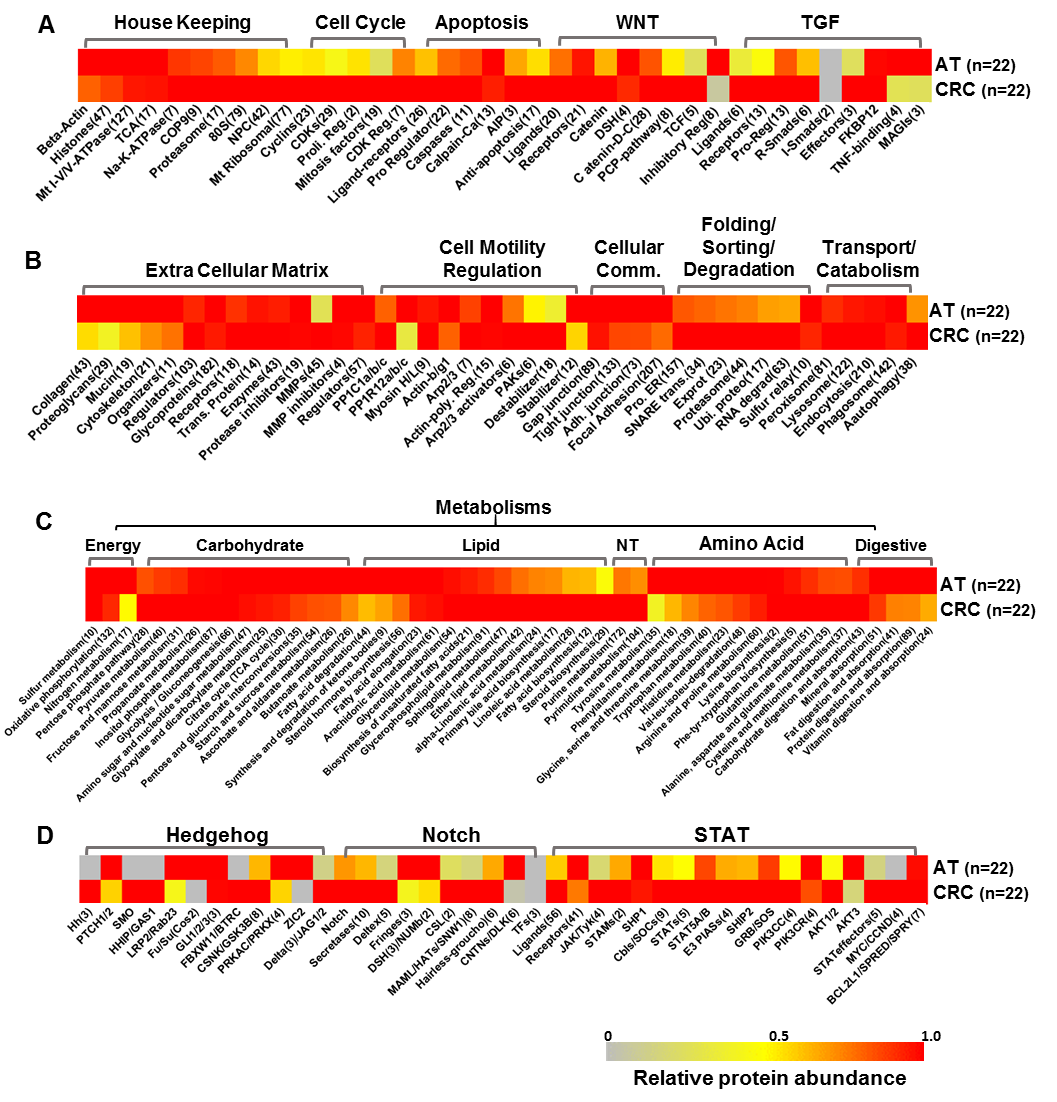
**

**Supplementary Fig. 3. The hallmarks of the proteome transition illustrated by the differential expression of POCs in analyzed signaling pathways or cellular processes.** Heat map panels A – I depict the comparison of the relative abundance of pathway protein ontology chains (POCs) at an average of 22 paired CRC and AT samples. The averages of 22 CRCs or 22 ATs are displayed as rows, and the POCs are displayed as columns. Pathways and the full name and data of POCs are listed in Table S4 and S9. The color key indicates the relative abundance of POCs (0 to 1.0) at an average (n=22) between CRC and AT. NT, nucleotide.

**Supplementary Figure 3 (continued)**

**
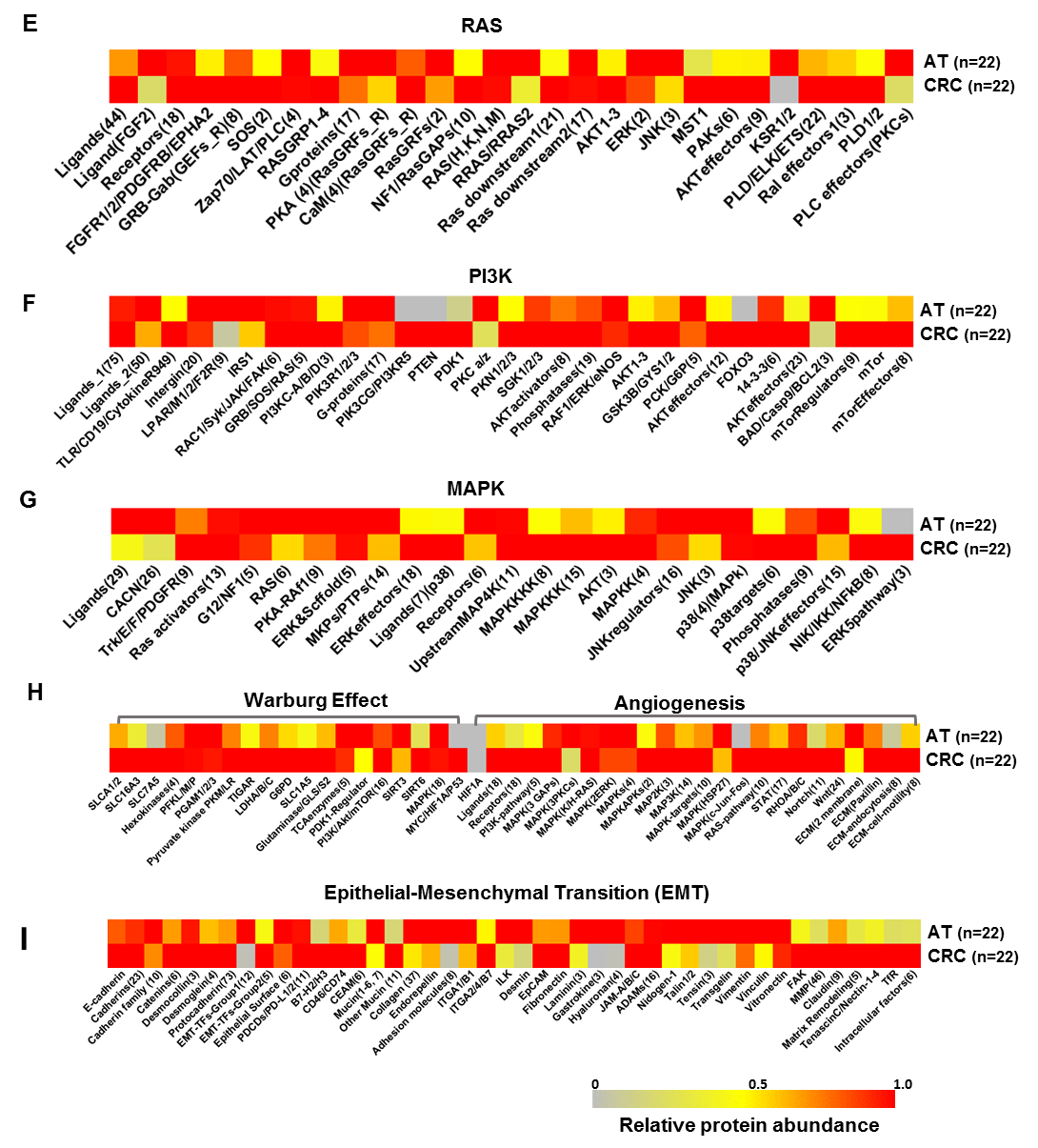
**

**Supplementary Figure 4**

**
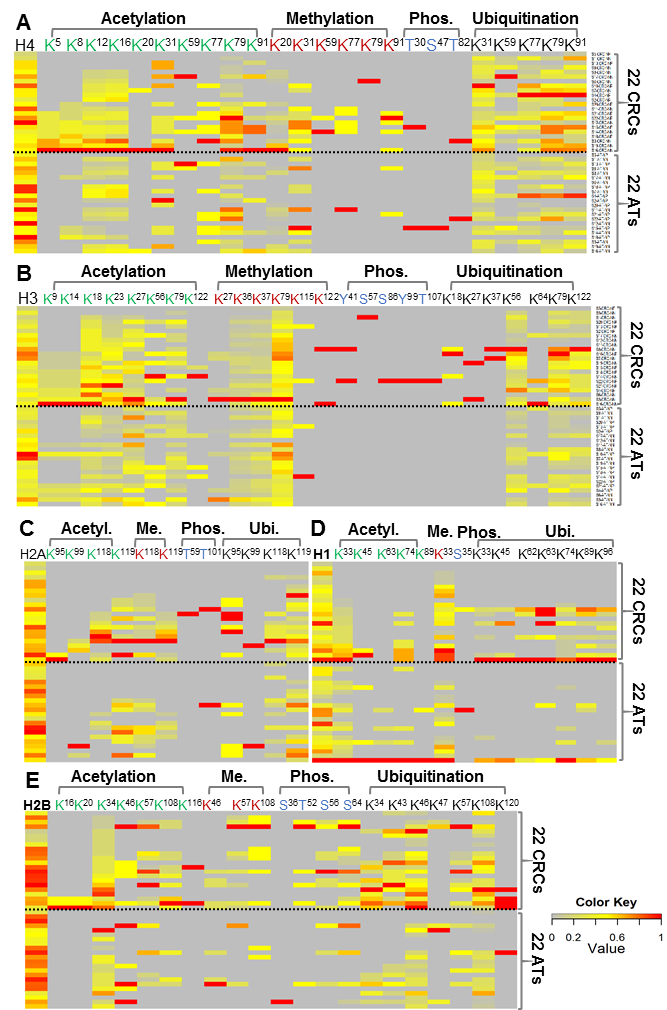
**

**Supplementary Fig. 4. Comparison of post-translational modifications on histone proteins between CRC and AT.** Approximately22CRCs and 22 ATs are displayed as rows, and the relative abundance of histone protein and relative abundance of modified sites are displayed as columns. **(A)** Histone H4 (left column) and all modified sites; **(B)** Histone H3 (left column) and all modified sites; **(C)** Histone H2A (left column) and all modified sites; **(D)** Histone H1 (left column) and all modified sites; **(E)** Histone H2B (left column) and all modified sites.The color key indicates the relative abundance of proteins or modification sites (0 to 1.0) across 44 samples (22 paired CRCs and ATs).

**Supplementary Figure 5**

**
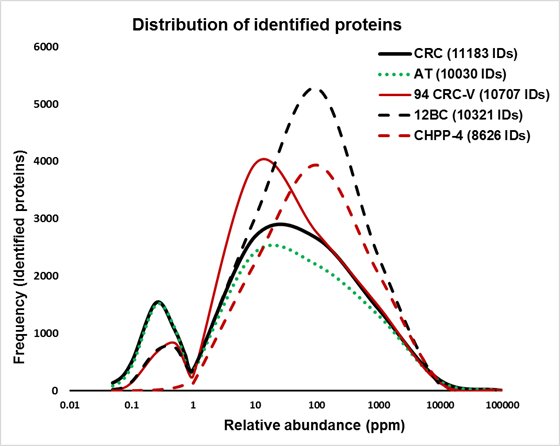
**

**Supplementary Fig. 5. Distributions of the protein populations identified by tandem mass spectrometry and quantified by spectral counting.** The graphs show normal distributions of protein populations identified by tandem mass spectrometry. The x-axis is the log scale of abundance (ppm), and the y-axis is the corresponding number of identified proteins. In this figure, five graphs represent five proteomes: the CRC proteome based on 22 CRC samples, the AT proteome based on 22 AT samples, the TCGA-CRC proteome based on 94 CRC samples from the TCGA colorectal cancer program, the TCGA breast cancer proteome based on 12 sets of MS raw files from the TCGA breast cancer program, and the CHPP cell line proteome based on 4 sets of MS raw files from CHPP program.

**Supplementary Figure 6**


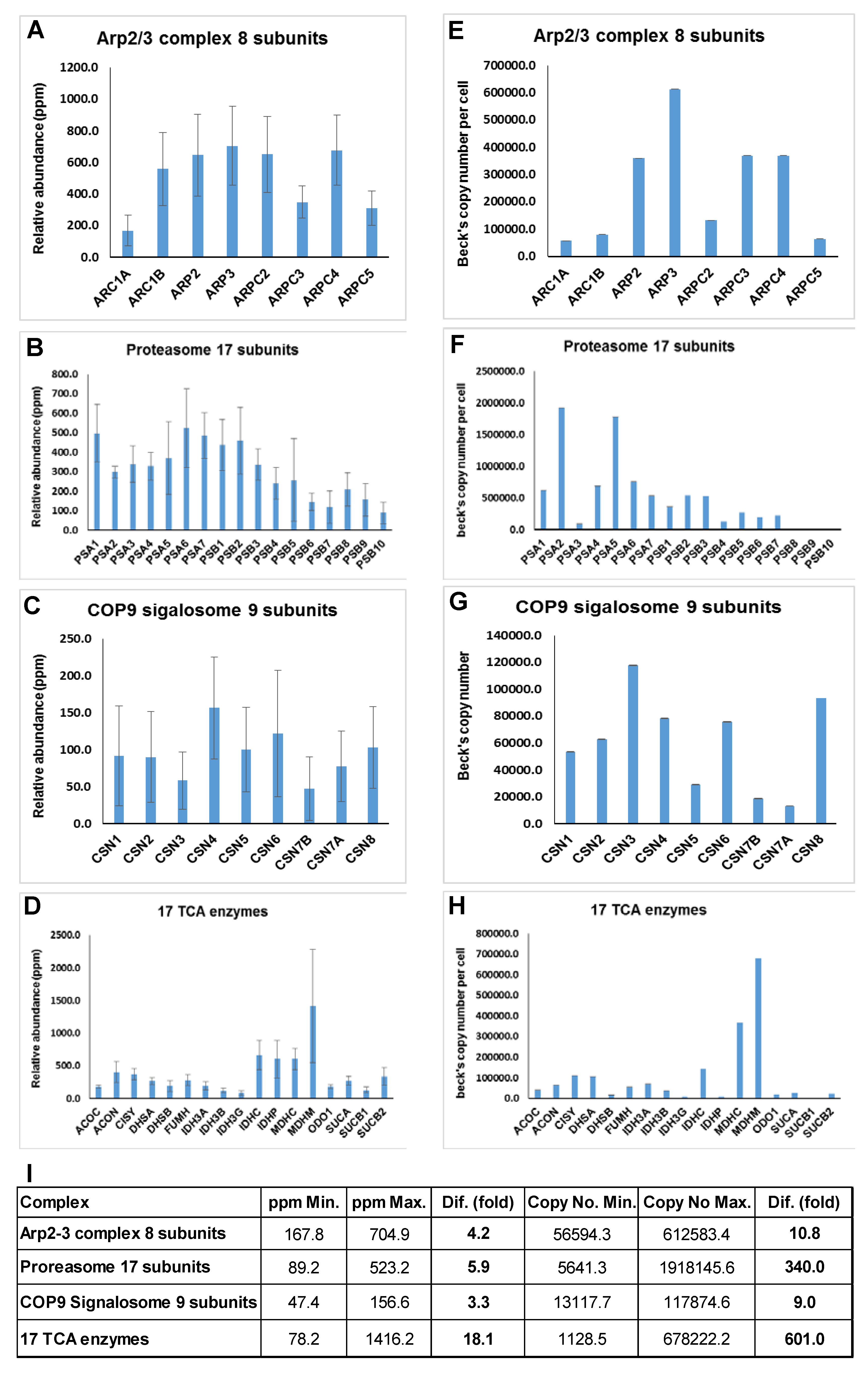


**Supplementary Fig. 6.Comparison of spectral counting based ppm quantitation with the Beck’s copy number quantitation.** The average abundance (ppm) of each subunit from four “housekeeping” protein complexes were based on 22 CRC samples, 22 AT samples, 94 TCGA CRC samples, 12 TCGA breast cancer samples, 4 raw datasets from CHPP program, and 3 mesenchymal stem cell (MSC) samples. Three proteome profiles from three lots of MSC were generated according to the same method described in Supplementary Methods. The ppm or Beck’s copy number for each subunit is presented in (**a**) or (**e**) for the Arp2/3 complex (8 subunits), in (**b**) or (**f**) for the Proteasome (17 subunits), in (**c**) or (**g**) for the COP9 signalosome (9 subunits), and in (**d**) or (**h**) for the 17 TCA enzymes. (**i**) Comparison of the dynamic range of abundances for four complexes. The Beck’s copy numbers were obtained from the article published by Beck et al^30^.

**Supplementary Figure 7**


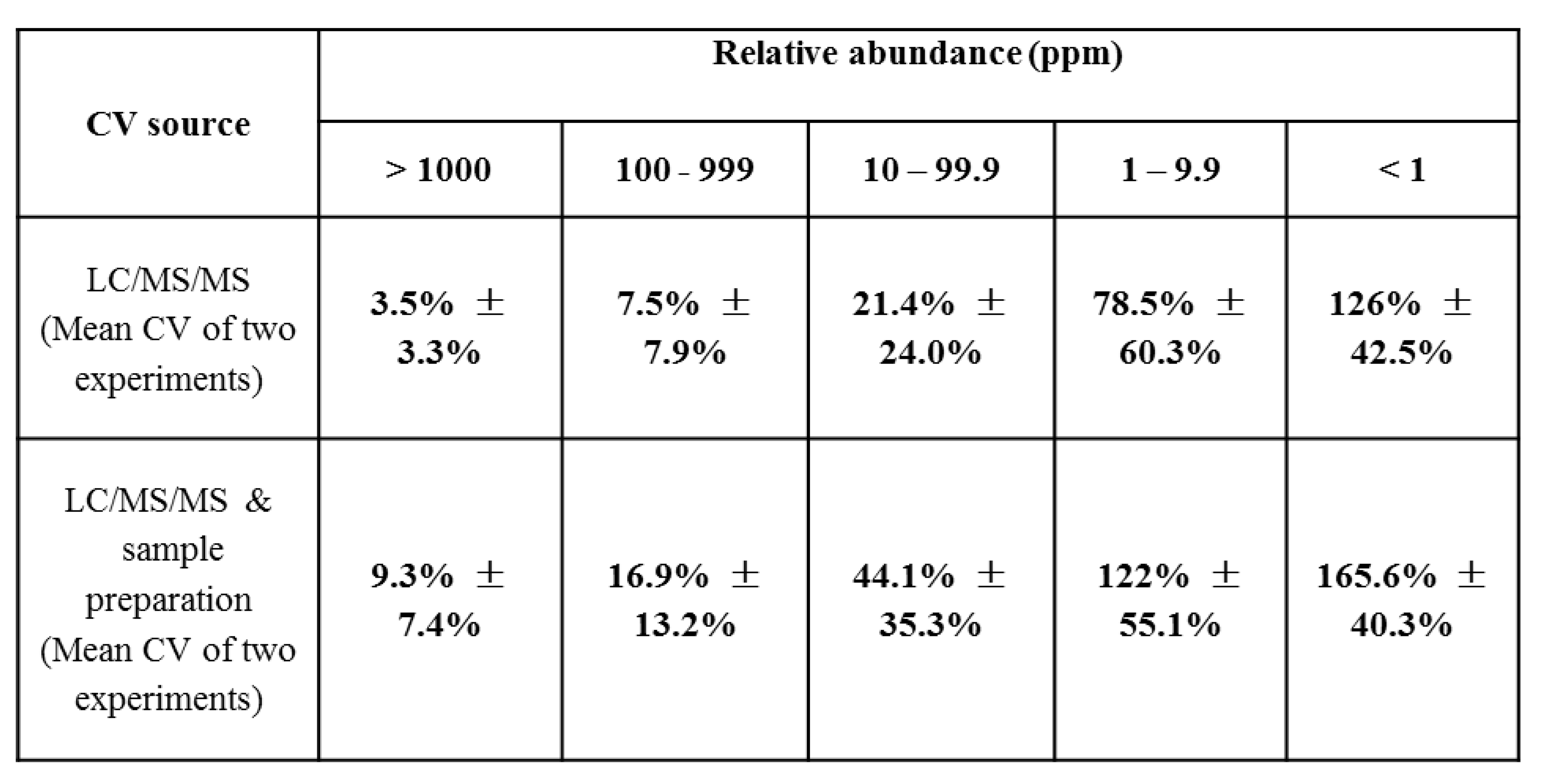


**Supplementary Fig. 7. Evaluation of the coefficient of variations of PSM counts based label-free quantification.** The CVs caused either by the LC-MS/MS system or by sample preparation were determined as described in the methods.

**Supplementary Table 1:** Sample index and clinical pathological records.

| **Patient No.** | **Biopsy No.** | **Tissue** | **Diagnosis** | **Location** | **Lymph node examined count** | **Lymphatic invasion** | **Pathologic stage** | **Protein extraction (mg/mL)** |
| --- | --- | --- | --- | --- | --- | --- | --- | --- |
| 1 | S1-CRC-NN | CRC | rectal adenocarcinoma | rectum | 12 | 0 | T2N0M0 | 9.79 |
| 2 | S1-AT-NN | AT | rectal adenocarcinoma | rectum | 12 | 0 | N/A | 9.32 |
| 3 | S2-CRC-NP | CRC | colon adenocarcinoma | sigmoid colon | 12 | 1 | T4N1M0 | 10.89 |
| 4 | S2-AT-NP | AT | colon adenocarcinoma | sigmoid colon | 12 | 1 | N/A | 5.89 |
| 5 | S3-CRC-NN | CRC | rectal adenocarcinoma | rectum | 17 | 0 | T2N0M0 | 9.08 |
| 6 | S3-AT-NN | AT | rectal adenocarcinoma | rectum | 17 | 0 | N/A | 9.87 |
| 7 | S4-CRC-NP | CRC | colon adenocarcinoma | ascending colon | 12 | 1 | T4N1M0 | 7.39 |
| 8 | S4-AT-NP | AT | colon adenocarcinoma | ascending colon | 12 | 1 | N/A | 9.07 |
| 9 | S5-CRC-NP | CRC | rectal adenocarcinoma | rectum | 17 | 2 | T4N1M0 | 12.97 |
| 10 | S5-AT-NP | AT | rectal adenocarcinoma | rectum | 17 | 2 | N/A | 11.81 |
| 11 | S6-CRC-NN | CRC | rectal adenocarcinoma | rectum | 13 | 0 | T2N0M0 | 10.66 |
| 12 | S6-AT-NN | AT | rectal adenocarcinoma | rectum | 13 | 0 | N/A | 11.38 |
| 13 | S7-CRC-NN | CRC | colon adenocarcinoma | descending colon | 13 | 0 | T3N0M0 | 10.58 |
| 14 | S7-AT-NN | AT | colon adenocarcinoma | descending colon | 13 | 0 | N/A | 6.55 |
| 15 | S8-CRC-NN | CRC | colon adenocarcinoma | sigmoid colon | 11 | 0 | T4N0M0 | 11.21 |
| 16 | S8-AT-NN | AT | colon adenocarcinoma | sigmoid colon | 11 | 0 | N/A | 9.14 |
| 17 | S9-CRC-NN | CRC | rectal adenocarcinoma | rectum | 8 | 0 | T2N0M0 | 13.21 |
| 18 | S9-AT-NN | AT | rectal adenocarcinoma | rectum | 8 | 0 | N/A | 9.68 |
| 19 | S10-CRC-NP | CRC | rectal adenocarcinoma | rectum | 14 | 4 | T3N2M0 | 9.94 |
| 20 | S10-AT-NP | AT | rectal adenocarcinoma | rectum | 14 | 4 | N/A | 7.38 |
| 21 | S11-CRC-NN | CRC | rectal adenocarcinoma | rectum | 14 | 0 | T3N0M0 | 9.14 |
| 22 | S11-AT-NN | AT | rectal adenocarcinoma | rectum | 14 | 0 | N/A | 8.45 |
| 23 | S12-CRC-NN | CRC | rectal adenocarcinoma | rectum | 14 | 0 | T2N0M0 | 9.54 |
| 24 | S12-AT-NN | AT | rectal adenocarcinoma | rectum | 14 | 0 | N/A | 9.17 |
| 25 | S13-CRC-NP | CRC | rectal adenocarcinoma | rectum | 22 | 7 | T3N2M0 | 12.33 |
| 26 | S13-AT-NP | AT | rectal adenocarcinoma | rectum | 22 | 7 | N/A | 10.32 |
| 27 | S14-CRC-NN | CRC | colon adenocarcinoma | transverse colon | 11 | 0 | T1N0M0 | 12.16 |
| 28 | S14-AT-NN | AT | colon adenocarcinoma | transverse colon | 11 | 0 | N/A | 13.42 |
| 29 | S15-CRC-NP | CRC | colon adenocarcinoma | sigmoid colon | 12 | 11 | T3N2M0 | 8.40 |
| 30 | S15-AT-NP | AT | colon adenocarcinoma | sigmoid colon | 12 | 11 | N/A | 5.44 |
| 31 | S16-CRC-NN | CRC | rectal adenocarcinoma | rectum | 14 | 0 | T2N0M0 | 11.79 |
| 32 | S16-AT-NN | AT | rectal adenocarcinoma | rectum | 14 | 0 | N/A | 9.07 |
| 33 | S17-CRC-NN | CRC | colon adenocarcinoma | sigmoid colon | 17 | 0 | T3N0M0 | 10.45 |
| 34 | S17-AT-NN | AT | colon adenocarcinoma | sigmoid colon | 17 | 0 | N/A | 8.93 |
| 35 | S18-CRC-NP | CRC | colon adenocarcinoma | ascending colon | 16 | 2 | T4N1M0 | 9.63 |
| 36 | S18-AT-NP | AT | colon adenocarcinoma | ascending colon | 16 | 2 | N/A | 6.75 |
| 37 | S19-CRC-NN | CRC | colon adenocarcinoma | sigmoid colon | 12 | 0 | T3N0M0 | 11.46 |
| 38 | S19-AT-NN | AT | colon adenocarcinoma | sigmoid colon | 12 | 0 | N/A | 8.90 |
| 39 | S20-CRC-NP | CRC | colon adenocarcinoma | transverse colon | 21 | 1 | T4N1M0 | 12.47 |
| 40 | S20-AT-NP | AT | colon adenocarcinoma | transverse colon | 21 | 1 | N/A | 9.85 |
| 41 | S21-CRC-NP | CRC | rectal adenocarcinoma | rectum | 20 | 2 | T3N1M0 | 9.16 |
| 42 | S21-AT-NP | AT | rectal adenocarcinoma | rectum | 20 | 2 | N/A | 7.81 |
| 43 | S22-CRC-NP | CRC | colon adenocarcinoma | sigmoid colon | 15 | 9 | T3N2M0 | 8.85 |
| 44 | S22-AT-NP | AT | colon adenocarcinoma | sigmoid colon | 15 | 9 | N/A | 7.03 |

**Supplementary Table 2:** Protein classification and coverage according to the UniProtKB human proteome database.

| **Classification (UniprotKB)** | **Classification (short name)** | **% Coverage** | **Protein (UniprotKB)** | **Protein from CRC&AT** | **Protein from CRC** | **Protein from AT** | **CRC total abundance (ave.) (ppm)** | **AT total abundance (ave.) (ppm)** | **% Change of abundance (CRCvsAT)** |
| --- | --- | --- | --- | --- | --- | --- | --- | --- | --- |
| **Molecular_Function (14420)** | **Molecular_Function (14420)** | 65.4% | 14420 | 9428 | 8639 | 7820 | 934526.0 | 935789.5 | -0.1% |
| binding (10752) | binding (10752) | 68.6% | 10752 | 7377 | 6804 | 6160 | 809820.2 | 814426.5 | -0.6% |
| catalytic activity (5312) | catalytic (5312) | 76.9% | 5312 | 4083 | 3832 | 3497 | 350618.3 | 340270.1 | 3.0% |
| signal transducer activity (1634) | signal transducer (1634) | 42.9% | 1634 | 701 | 579 | 534 | 18270.4 | 18592.5 | -1.7% |
| receptor activity (1546) | receptor (1546) | 40.2% | 1546 | 621 | 506 | 451 | 14375.6 | 14230.3 | 1.0% |
| transporter activity (1185) | transporter (1185) | 64.1% | 1185 | 760 | 678 | 644 | 91886.9 | 103287.5 | -11.0% |
| nucleic acid binding transcription factor activity (1046) | NA binding TF (1046) | 44.9% | 1046 | 470 | 384 | 317 | 12672.4 | 8250.9 | 53.6% |
| enzyme regulator activity (861) | enzyme regulator (861) | 72.9% | 861 | 628 | 588 | 532 | 67812.9 | 60533.7 | 12.0% |
| structural molecule activity (619) | structural molecule (619) | 80.3% | 619 | 497 | 480 | 464 | 218451.4 | 221320.6 | -1.3% |
| protein binding transcription factor activity (535) | PB TF (535) | 64.5% | 535 | 345 | 319 | 270 | 17576.0 | 11977.0 | 46.7% |
| guanyl-nucleotide exchange factor activity (179) | guanyl-NT EF (179) | 78.8% | 179 | 141 | 126 | 112 | 963.2 | 879.8 | 9.5% |
| electron carrier activity (100) | electron carrier (100) | 90.0% | 100 | 90 | 87 | 88 | 14658.9 | 15053.9 | -2.6% |
| channel regulator activity (97) | channel regulator (97) | 66.0% | 97 | 64 | 59 | 51 | 4795.6 | 4828.9 | -0.7% |
| antioxidant activity (65) | antioxidant (65) | 90.8% | 65 | 59 | 58 | 55 | 26656.0 | 31260.2 | -14.7% |
| receptor regulator activity (41) | receptor regulator (41) | 63.4% | 41 | 26 | 24 | 20 | 685.3 | 652.3 | 5.0% |
| translation regulator activity (33) | translation regulator (33) | 69.7% | 33 | 23 | 20 | 20 | 5880.3 | 3874.9 | 51.8% |
| chemoattractant activity (22) | chemoattractant (22) | 40.9% | 22 | 9 | 8 | 7 | 4095.4 | 3444.1 | 18.9% |
| chemorepellent activity (8) | chemorepellent (8) | 87.5% | 8 | 7 | 7 | 4 | 660.6 | 995.4 | -33.6% |
| morphogen activity (6) | morphogen (6) | 33.3% | 6 | 2 | 2 | 1 | 12.5 | 11.9 | 5.2% |
| metallochaperone activity (5) | metallochaperone (5) | 100.0% | 5 | 5 | 5 | 3 | 774.6 | 777.1 | -0.3% |
| protein tag (2) | protein tag (2) | 50.0% | 2 | 1 | 1 | 1 | 64.2 | 11.7 | 447.9% |
| nutrient reservoir activity (1) | nutrient reservoir (1) | 100.0% | 1 | 1 | 1 | 1 | 0.1 | 0.2 | -42.2% |
| **Cellular_Component (17465)** | **Cellular_Component (17465)** | 63.8% | 17465 | 11143 | 10144 | 9190 | 992912.5 | 994373.0 | -0.1% |
| cell (15347) | cell (15347) | 66.2% | 15347 | 10159 | 9308 | 8435 | 955710.2 | 949500.6 | 0.7% |
| cell part (15346) | cell part (15346) | 66.2% | 15346 | 10159 | 9308 | 8435 | 955710.2 | 949500.6 | 0.7% |
| organelle (12291) | organelle (12291) | 71.3% | 12291 | 8758 | 8130 | 7409 | 934105.4 | 917138.0 | 1.9% |
| membrane (8792) | membrane (8792) | 59.9% | 8792 | 5270 | 5206 | 4836 | 583104.8 | 561817.2 | 3.8% |
| membrane part (6351) | membrane part (6351) | 58.6% | 6351 | 3724 | 3304 | 3054 | 219979.5 | 199188.4 | 10.4% |
| organelle part (5982) | organelle part (5982) | 77.8% | 5982 | 4654 | 4421 | 4014 | 670382.9 | 648336.8 | 3.4% |
| extracellular region (4480) | extracellular region (4480) | 72.2% | 4480 | 3234 | 3020 | 2869 | 734646.9 | 775122.0 | -5.2% |
| macromolecular complex (4440) | macromol. complex (4440) | 77.1% | 4440 | 3422 | 3241 | 2952 | 586719.4 | 574459.6 | 2.1% |
| extracellular region part (3633) | EC region part (3633) | 79.8% | 3633 | 2900 | 2741 | 2634 | 755842.3 | 792884.7 | -4.7% |
| membrane-enclosed lumen (2607) | membrane lumen (2607) | 80.2% | 2607 | 2090 | 1994 | 1764 | 299828.0 | 282500.0 | 6.1% |
| cell junction (1056) | cell junction (1056) | 76.1% | 1056 | 804 | 740 | 710 | 159072.3 | 162162.0 | -1.9% |
| synapse (553) | synapse (553) | 63.8% | 553 | 353 | 296 | 305 | 27969.1 | 32520.4 | -14.0% |
| synapse part (389) | synapse part (389) | 59.9% | 389 | 233 | 191 | 199 | 17956.9 | 20199.3 | -11.1% |
| extracellular matrix (381) | ECM (381) | 71.1% | 381 | 271 | 241 | 229 | 35762.8 | 48699.4 | -26.6% |
| extracellular matrix part (121) | ECM part (121) | 85.1% | 121 | 103 | 98 | 93 | 16449.6 | 22742.1 | -27.7% |
| collagen trimer (87) | collagen trimer (87) | 75.9% | 87 | 66 | 55 | 55 | 4360.8 | 8933.4 | -51.2% |
| mitochondrial nucleoid (40) | mt nucleoid (40) | 97.5% | 40 | 39 | 39 | 38 | 12362.3 | 10420.1 | 18.6% |
| virion part (35) | virion part (35) | 22.9% | 35 | 8 | 7 | 5 | 1.6 | 1.0 | 60.7% |
| **Biological_process (16149)** | Biological_Process (16149) | 65.2% | 16149 | 10533 | 9615 | 8696 | 956865.7 | 957126.8 | 0.0% |
| cellular process (13818) | cellular process (13818) | 65.8% | 13818 | 9092 | 8321 | 7534 | 870698.1 | 854703.6 | 1.9% |
| single-organism process (12514) | single-organism (12514) | 66.8% | 12514 | 8361 | 7637 | 7002 | 871709.9 | 886959.6 | -1.7% |
| biological regulation (10602) | biological regulation (10602) | 62.6% | 10602 | 6637 | 6001 | 5444 | 632625.6 | 639833.7 | -1.1% |
| metabolic process (9899) | metabolic process (9899) | 70.4% | 9899 | 6965 | 6465 | 5826 | 690257.4 | 667823.8 | 3.4% |
| response to stimulus (7383) | response to stimulus (7383) | 64.3% | 7383 | 4749 | 4318 | 3949 | 574496.3 | 599472.3 | -4.2% |
| multicellular organismal process (6111) | multicellular organismal(6111) | 61.6% | 6111 | 3765 | 3367 | 3102 | 426029.1 | 471793.3 | -9.7% |
| signaling (5129) | signaling (5129) | 59.8% | 5129 | 3065 | 2743 | 2541 | 291594.7 | 284432.2 | 2.5% |
| developmental process (4837) | developmental(4837) | 65.8% | 4837 | 3182 | 2853 | 2615 | 311714.8 | 322380.6 | -3.3% |
| localization (4477) | localization (4477) | 72.5% | 4477 | 3244 | 3009 | 2809 | 458122.2 | 460182.4 | -0.4% |
| cellular component organization or biogenesis (4473) | cellular component OB(4473) | 76.6% | 4473 | 3427 | 3219 | 2928 | 468922.0 | 459063.0 | 2.1% |
| immune system process (2188) | immune system (2188) | 70.5% | 2188 | 1542 | 1434 | 1335 | 252796.6 | 249842.0 | 1.2% |
| multi-organism process (2093) | multi-organism (2093) | 70.1% | 2093 | 1467 | 1373 | 1230 | 245586.9 | 201052.9 | 22.2% |
| reproductive process (1156) | reproductive process (1156) | 60.6% | 1156 | 700 | 611 | 541 | 50202.7 | 43829.9 | 14.5% |
| locomotion (1099) | locomotion (1099) | 67.8% | 1099 | 745 | 686 | 641 | 101051.8 | 102743.6 | -1.6% |
| reproduction (902) | reproduction (902) | 62.9% | 902 | 567 | 510 | 425 | 37981.3 | 32702.8 | 16.1% |
| biological adhesion (854) | biological adhesion (854) | 70.8% | 854 | 605 | 556 | 502 | 82819.4 | 97433.5 | -15.0% |
| growth (374) | growth (374) | 62.7% | 374 | 234 | 204 | 187 | 7632.0 | 6614.2 | 15.4% |
| rhythmic process (244) | rhythmic process (244) | 61.9% | 244 | 151 | 135 | 120 | 18459.8 | 20139.9 | -8.3% |
| hormone secretion (71) | hormone secretion (71) | 47.9% | 71 | 34 | 31 | 24 | 4300.1 | 3945.8 | 9.0% |
| cell killing (46) | cell killing (46) | 69.6% | 46 | 32 | 31 | 29 | 23223.8 | 25475.5 | -8.8% |
| biological phase (24) | biological phase (24) | 66.7% | 24 | 16 | 12 | 12 | 1229.5 | 1337.7 | -8.1% |

**Supplementary Datasets:**

**Supplementary Dataset 1:** Report of the protein identification of proteomes of 44 samples.

**Supplementary Dataset 2:** Quantification of 44 proteome profiles by spectral counting and statistical analysis. Note: If any protein was not identified in AT or in CRC, the ratio of CRC versus AT was defined as 1,000 or 0.001, respectively.

**Supplementary Dataset 3:** The list of proteins in all pathways and their quantitation in CRCs and ATs. The average of relative protein abundances was calculated based on 22 CRCs or 22 ATs.

**SupplementaryDataset 4:**(A) Summary of the evaluation of the proteomes of 22 CRCs and 22 ATs by 10 “housekeeping” protein complexes.(B) Summary of the evaluation of 12 TCGA breast cancer proteomic profiles by 10 “housekeeping” protein complexes.(C) Summary of the evaluation of 94 TCGA CRC proteomic profiles by 10 “housekeeping” protein complexes.

**SupplementaryDataset 5:** Annotation of ranked 1,640 CRC proteomic signatures.

**SupplementaryDataset 6**: (A) 715 CRC protein signatures across 44 samples, (B) 567 proteins (listed in Sadanandam 786 CRC assigner) identified across 44 samples, (C) 102 proteins (listed in Melo CCSs 146 gene classifier) identified across 44 samples, and (D) 100 proteins (listed in Vogelstein 125 tumor driver genes) identified across 44 samples.

**SupplementaryDataset 7: (A)** Quantification of the 12 TCGA breast cancer proteomic profiles by spectral counting.**(B)**Quantification of the 94 TCGA CRC proteomic profiles by spectral counting.

***Supplementary Data analysis***

**LC/MS/MS data analysis and protein quantification.**

To quantify the changes in the CRC proteome, paired tumor and AT samples were processed and fractionated at the protein level by SDS–polyacrylamide gel electrophoresis and at the peptide level by basic reversed-phase liquid chromatography and analyzed on a high-resolution Fourier-transform mass spectrometer (Q-Exactive Orbitrap). Approximately 44 proteomic profiles were generated by analyzing 22 paired CRC and AT samples (Supplementary Table 1, Supplementary Dataset 1). The relative abundance for each identified protein in each proteome profile was calculated using the normalized spectral abundance factors (NSAFs) method ^34,35^. To quantitatively describe the relative abundance, ppm (parts per million) was chosen as the unit with a total 1,000,000 ppm assigned to each proteome profile and was calculated based on its normalized NSAF. The Average, STDEV, CV and paired T-test (p-values) were calculated using Microsoft Excel. The quantification data was listed in Dataset 2. To evaluate this method two sets of public available MS raw data files (Ninety-four sets of MS raw data files from The Cancer Genome Atlas (TCGA) CRC cancer program and 12 sets of MS raw datasets from the TCGA breast cancer program were downloaded from <https://cptac-data-portal.georgetown.edu/cptacPublic/>) were analyzed using the same method. The results were listed in Dataset 7A and Dataset 7B, respectively.

To evaluate the ppm quantification method, we compared the ppm variation ranges among of subunits of four complexes (Arp2/3 complex COP9 complex, 17 proteasome subunits, and 17 TCA enzymes) with published Beck’s copy numbers data^36^. As shown in Supplementary Fig. 5, the dynamic range of relative abundance among the members of a complex quantified using ppm was considerably reduced compared to that of Beck’s copy number. The dynamic range between the minimum and maximum for the four tested complexes exhibited 4- to 19-fold differences according to spectrum count-based measurements, whereas 9- to 600-fold differences were noted according to the published Beck’s copy number. This comparison indicated that the relative protein abundance measured based on spectrum count quantification would be closer to the real situation and that the housekeeping protein complexes could be used as the parameter to evaluate the “quality” of a proteome profile generated thereof.

We also assessed the distribution of quantified protein population according to their relative abundance. The distribution of identified proteins per concentration range was analyzed using the Excel-histogram function. The average abundance for each identified protein was calculated as described above. The distribution of all identified 12,380 proteins was normal, with a major peak and a minor peak representing two populations. The major peak represented 62% (CRC) and 60% (AT) of identified proteins with a relative abundance greater than 1 ppm, and the minor peak represented approximately 38% (CRC) and 40% (AT) of identified proteins with an abundance less than 1 ppm. The majority of proteins in the minor peak were randomly identified with one or a few PSM across 22 CRC samples or 22 AT samples (Supplementary Dataset 1). To further evaluate the method, 94 sets of MS raw profiles from the TCGA-CRC cancer program, 12 sets of MS raw datasets from the TCGA-breast cancer program, and 4 sets of MS raw datasets from CHPP program were analyzed. The distributions of identified proteins in these studies exhibited the same normal distribution patterns (Supplementary Fig. 4).

**Evaluation of the “quality/integrity” of proteome profiles.**

Due to instrument limitations and the wide dynamic range of protein abundances, the most current LC/MS/MS settings were unable to recover the entire proteome, particularly the lowest abundance proteins in one experiment. Hence, development of an approach allowing evaluation of the integrity of a set of proteome profiles in an unbiased way is highly desirable. To achieve this goal, we focused on ten groups of well-characterized “housekeeping” protein complexes with the assumption that these proteins are essential for all live cells and that their detections would serve as an internal quality control for a set of proteomic profiles. Ten groups of well-known “housekeeping” protein complexes consisting of 444 proteins, including 359 unique proteins and 85 isoforms or subtypes (Supplementary Dataset 3), included the Arp2/3 complex (8 subunits plus alpha and beta actins), 86 (79 and 7 isoforms) cellular (60S and 40S) ribosomal proteins, 77 mitochondrial (28S and 39S) ribosomal proteins, nuclear pore complex 42 (38 subunits, GTP-binding nuclear protein Ran, Ran GTPase-activating protein 1 (RAGP1), Ran-specific GTPase-activating protein (RANG), Ran-binding protein 3 (RANB3)), 5 histones (H1 (9 subtypes), H2A (17 subtypes), H2B (17 subtypes), H3 (5 subtypes) and H4), proteasome complex (17 subunits), COP9 signalosome complex (9 subunits), TCA enzymes (17 key enzymes), mitochondrial respiratory chain complexes I -V (102 subunits), V-type proton (ATPase complex, 14 subunits consisting 24 isoforms), and Na+/K+-ATPase (sodium-potassium pump, 2 subunits, 7 isoforms). A score (0 to 100) was assigned based on the percentage of the 444 “housekeeping” proteins identified in a given profile. On average, 44 proteome profiles generated in this study were scored at 92, suggesting a consistent quality with all these profiles. To demonstrate the feasibility of this evaluation method, we assessed two sets of publically available MS raw data files (http://proteomics.cancer.gov/). One set of 94 MS raw data files (94 CRC samples) from the TCGA-CRC cancer program was scored at an average of 80.3. An additional set of 12 MS raw data files from the TCGA-breast cancer program was scored at an average of 98.5 (Supplementary Dataset 4).

**Evaluation of the reliability of label-free based quantitative proteomic profiling workflow.**

The standardized spectral counting-based label-free quantitative proteomics workflow (Supplementary Fig. 1) described above was evaluated by examining the coefficients of variation (CVs) of quantified among experiments. The CVs caused either by the LC-MS/MS system or by sample preparation were determined. The CVs caused by the inherent LC-MS/MS system (system error) included the nanoLC separation and the mass spectrometry measurement stability along with any potential inconsistencies related to the bioinformatic extractions of peptides by the proteome discoverer software. To define the CV caused by different LC/MS/MS runs, a set of 16 fractions prepared from a sample were run sequentially in duplicate. Two independent experiments were performed. As shown in Supplementary Fig. 6, the CV caused by the LC-MS system varied in reference to the relative concentration of proteins identified. The LC-MS/MS system caused the CV for the higher abundance proteins to be considerably reduced compared with the CV of the less abundant proteins. For example, the CV was less than 5% if the relative abundance of identified proteins was more than 1,000 ppm, but the CV was near 78% if the relative concentration of identified proteins was between 1 to 10 ppm due to the limitation of the mass spectrometer in detecting low-concentration peptides from a mixture. The average CV for all identified proteins between two independent analyses was 48% ± 27%. Because the lowest abundant proteins were mostly identified by either one independent analysis, the average CV for these proteins (<1 ppm) was increased (128% ± 40%). The average CV for all proteins with >1 ppm, which represented greater than 90% of identified proteins, was 28% ± 24%. Next, we evaluated the variation caused by sample processing by analyzing the same sample processed in triplicate. The average CV for proteins >1 ppm was 42.2% ± 27.5%. Given that system variation was inherent and independent of the sample, the CV caused by sample processing could be deduced by subtracting the system CV. Manual sample processing caused an average of 15% CV. In all analyses, the CV values and change patterns were similar, indicating that the proteomic workflow was reliable and repeatable.

**Pathway analysis.**

Biological information flow from the DNA/genome to protein/proteome automatically regulates cell living status in response to internal or external signals. Cancer genome, in one aspect, illustrated by mutations and instability shapes the current molecular foundation of tumorigenesis. As Vogelstein summarized the accumulation of mutations from 125 tumor driver genes (oncogenes and tumor suppressor genes) discovered so far promote the tumorigenesis. The tumor phenotype, in another aspect, described as hallmarks of cancer (acquired biological capabilities) by Hanahan and Weinberg, demonstrates sustaining proliferative signaling, evading growth suppressors, resisting cell death, enabling replicative immortality, inducing angiogenesis, activating invasion and metastasis inflammation, reprogramming of energy metabolism, and evading immune destruction. As the link from the gene to the phenotype, proteins play the key roles in orchestrating all biological processes, ranging from central metabolism to cell structure, maintenance, and replication. It is speculated that all biological processes (on/off) are regulated and operated by changing the abundance of their players, the proteins. The regulation of different cellular functions has been categorized into a number of pathways, such as the Wnt signaling pathway and the TGF signaling pathway. In each pathway, the components are generally named according to their function, including ligands, receptors, activating regulators, inhibitory regulators, and effectors. To measure the activation strength of a pathway, the protein molecules that belong to ligands, receptors, activating regulators, or inhibitory regulators were grouped as the pathway protein ontology chain (POC), and their relative abundances (ppm) were summed. Based on the summed abundance of each POC, the activation strength or activation status of a pathway could be compared between two proteome profiles. As demonstrated in Fig. 2A, Fig. 3 and SupplementaryFig. 2A significant pattern of changes in protein abundance was uncovered in this study using this novel pathway analysis approach.The pattern in tumor tissues demonstrates that 1) reduction in protein abundance of inhibitory regulators but increasing of activating regulators in key signal pathways, a significant elevation of proteins responsible for chromatin modification, gene expression and DNA replication and damage repair, and a decreased expression of proteins responsible for core extracellular matrix architectures. We name this global change pattern in protein abundance occurred in human colorectal cancer as“**tumor proteomic transition phenotype**”, which could explain how the tumor cells secure the selective growth advantages. This new concept is of considerable biological interest and could provide insight into understanding of molecular mechanisms of tumor genesis.

**Publically available MS raw data files used to evaluate the methods.**

Ninety-four sets of MS raw data files from The Cancer Genome Atlas (TCGA) CRC cancer program and 12 sets of MS raw datasets from the TCGA breast cancer program were downloaded from https://cptac-data-portal.georgetown.edu/cptacPublic/. Four sets of MS raw datasets from the CHPP proteome were downloaded from <http://dx.doi.org/10.6019/PXD000529/>.
